# Musashi2 contributes to the maintenance of CD44v6+ liver cancer stem cells via notch1 signaling pathway

**DOI:** 10.1186/s13046-019-1508-1

**Published:** 2019-12-30

**Authors:** Xiju Wang, Ronghua Wang, Shuya Bai, Si Xiong, Yawen Li, Man Liu, Zhenxiong Zhao, Yun Wang, Yuchong Zhao, Wei Chen, Timothy R. Billiar, Bin Cheng

**Affiliations:** 1 Department of Gastroenterology and Hepatology, Tongji Hospital, Tongji Medical College, Huazhong University of Science and Technology, Wuhan, People’s Republic of China 430030; 2 Department of Surgery, University of Pittsburgh School of Medicine, Pittsburgh, PA 15213 USA

**Keywords:** CD44 variant exon 6, Musashi2, Notch1 signaling pathway, Liver cancer stem cells, Hepatocellular carcinoma

## Abstract

**Background:**

Liver cancer stem cells (LCSCs) contribute to hepatocellular carcinoma (HCC) development, metastasis, and drug resistance. MSI2 and Notch1 signaling are involved in the maintenance of CSCs. However, it is unknown whether MSI2 and Notch1 are involved in the maintenance of CD44v6+ LCSCs. Therefore, we investigated the clinical significance and function of MSI2 and its relationship with Notch1 signaling in the maintenance of stemness properties in CD44v6+ LCSCs.

**Methods:**

The expression of MSI2 and CD44v6 were detected by fresh specimens and a HCC tissue microarray. The tissue microarray containing 82 HCC samples was used to analyze the correlation between CD44v6 and MSI2. CD44v6+/− cells were isolated using microbeads sorting. We explored the roles of MSI2 and Notch1 signaling in CD44v6+ LCSCs by sphere formation assay, transwell assay, clone formation assay in vitro, and xenograft tumor models in vivo. A Notch RT^2^ PCR Array, Co-immunoprecipitation, and RNA-immunoprecipitation were used to further investigate the molecular mechanism of MSI2 in activating Notch1 signaling.

**Results:**

Here, we found MSI2 expression was positively correlated with high CD44v6 expression in HCC tissues, and further correlated with tumor differentiation. CD44v6+ cells isolated from HCC cell lines exhibited increased self-renewal, proliferation, migration and invasion, resistance to Sorafenib and tumorigenic capacity. Both MSI2 and Notch1 signaling were elevated in sorted CD44v6+ cells than CD44v6- cells and played essential roles in the maintenance of stemness of CD44v6+ LCSCs. Mechanically, MSI2 directly bound to Lunatic fringe (LFNG) mRNA and protein, resulting in Notch1 activation.

**Conclusions:**

Our results demonstrated that MSI2 maintained the stemness of CD44v6+ LCSCs by activating Notch1 signaling through the interaction with LFNG, which could be a potential molecular target for stem cell-targeted therapy for liver cancer.

## Background

Hepatocellular carcinoma (HCC) is ranked as the sixth most common neoplasm and the third leading cause of cancer deaths [1]. Accumulating evidence has demonstrated that liver cancer stem cells (LCSCs), which possess the capacity of tumor initiation, self-renewal, metastasis and chemotherapy resistance contribute to the recurrence, metastasis, chemoradiotherapy resistance of HCC [2–5]. However, the mechanism of how LCSCs contribute to the progression of HCC remain to be elucidated. Enhancing the understanding of the molecular mechanism of LCSCs in cancer progression may promote the development of effective stem cell-targeted therapy and improve the prognosis of patients with HCC.

CSCs can be identified by cancer stem cell markers, and CD44 variant exon 6 (CD44v6) has been identified as one of the important markers of CSCs in many malignant tumors [6, 7]. CD44v6 is a variant exon of CD44 and CD44 has been identified as one of the important markers of LCSCs [8, 9]. CD44 is encoded by a highly conserved gene. Its pre-messenger RNA can be alternatively spliced into mature mRNAs which encode several isoforms, including ten standard exons, and the sixth variant exon encodes CD44v6 [8]. In our study, we confirmed that CD44v6+ cells exhibited increased self-renewal ability, proliferation, migration and invasion, resistance to Sorafenib and tumorigenic capacity than CD44v6- cells, indicating that CD44v6 could be a marker of LCSCs. However, the molecular mechanism of CD44v6 leading to HCC is not fully understood. Understanding the mechanism regulating the CD44v6+ LCSCs is vital for targeting LCSCs.

Musashi2 (MSI2) is a member of Musashi RNA-binding protein, which is a regulator of asymmetric division in *Drosophila* and is associated with stem and progenitor cells [10, 11]. MSI2 has been widely studied in hematopoietic malignancies, which promotes hematologic malignancies progression through activating Notch signaling by translational repression of Numb endocytic adaptor protein (Numb) [11–13]. In solid tumors, MSI2 has been shown to promote non-small cell lung cancer (NSCLC) metastasis via TGF-β signaling [14], and promote pancreatic cancer development and drug resistance [15, 16]. Previously, the studies of He and Wang et al. reported that MSI2 promotes progression and invasion in HCC via epithelial-mesenchymal transition and the Wnt/β-catenin pathway [17, 18]. Although significant progress has been made in understanding the contribution of MSI2 to malignancies, the functional contribution of MSI2 in LCSCs, especially in CD44v6+ LCSCs, is not known.

Notch signaling pathway is an evolutionarily highly conserved signaling, which is activated when the receptor interacts with the ligand, regulates CSCs proliferation, self-renewal, differentiation, angiogenesis, and migration [19–23]. The ligand-mediated Notch activation is modulated by fringe family of β3 N-acetylglucosaminyl-transferases, including Lunatic fringe (LFNG). And the activation of Notch could be regulated by LFNG on *O*-linked glycosylation of Epidermal Growth Factor (EGF) repeats in the Notch extracellular domain [24]. Our previous studies demonstrated that Notch signaling is elevated in LCSCs and act as key signaling to promote stem cell properties in HCC [25–27]. This central role of Notch in LCSC maintenance lead us to hypothesize the MSI2 and LFNG would be involved in the LCSC function in HCC.

In this study, we demonstrated that MSI2 was highly expressed in human HCC tissues and correlated with poor tumor differentiation, and poor prognosis. Importantly, we found the expression of MSI2 was positively correlated with that of CD44v6. Furthermore, MSI2 was elevated in isolated CD44v6+ LCSCs and played a crucial role in the maintenance of self-renewal, migration, invasion in vitro and tumorigenic capacity in vivo of CD44v6+ LCSCs. Mechanistic studies linked MSI2 to LFNG expression, which in turn upregulated Notch1 receptor expression and activation to maintain CD44v6+ LCSCs. These studies identify a previously undescribed relationship between MSI2 and LNFG signaling with Notch1 activation in LCSCs.

## Materials and methods

### Human HCC specimens

Two independent cohorts of HCC patients were enrolled in this study. To investigate the expression of CD44v6 and MSI2 in human HCC tissues, 28 tumorous and peri-tumoral samples were collected for western blot analysis (Cohort 1). Samples were collected from HCC patients undergoing curative resection in Tongji Hospital, Tongji Medical College, Huazhong University of Science and Technology (HUST, Wuhan, China). All specimens were collected immediately after resection and stored at − 80 °C. To evaluate the prognostic role of CD44v6 and MSI2 in HCC, Tissue microarrays (TMAs) containing 82 pairs of human HCC tissues specimens and adjacent liver specimens were collected for Immunohistochemistry (IHC). Samples from HCC patients underwent resection in Tongji Hospital. The diagnosis of HCC was verified by pathological outcomes. Clinical data of those specimens were recorded without patient identification. This project was approved by the ethics committee of Tongji Hospital, Tongji Medical College, Huazhong University of Science and Technology (HUST, Wuhan, China).

### Immunohistochemistry (IHC)

IHC staining with antibodies against CD44v6 (Abcam, ab78960), MSI2 (Abcam, ab76148) was performed to detect protein expression levels. The intensity of staining was scored on a scale as negative (0, no staining), weak (1, light yellow), moderate (2, brown), or strong (3, brown red). The extent of the staining was evaluated according to the percentage of positive areas of cells in relation to the whole area, was scored on a scale of 0–4, 0 (0), 1 (1–25%), 2 (26–50%), 3 (51–75%), and 4 (76–100%). Protein expression levels (range 0–12) were calculated by multiplying the staining intensity and positive staining score. Then, we divided the patients into two groups (grade<6, low expression; grade ≥ 6, high expression) and performed survival analysis. Assessment of IHC staining scores was independently performed by two pathologists (Dr. Yaqi Duan and Dr. Xi Wang, Department of Pathology, Tongji Hospital, Tongji Medical College, Huazhong University of Science and Technology), who were blinded to the clinical data. Examples of the staining intensity grades were shown in Additional file 1: Figure S1B-S1D.

### Immunofluorescence

Cells or tissue sections were fixed in 4% paraformaldehyde. After washing 3 times with PBS for 3 min each time, the samples were incubated in 10% normal donkey serum in PBS for 20 min, and then incubated with MSI2 (Abcam, ab76148), CD44v6 (Abcam, ab78960) or Notch1 (Abcam, ab52627) primary antibodies in PBS at 4 °C for overnight. Incubated the samples with fluorochrome-conjugated secondary antibodies (1:400, Alexa Fluor®488 donkey anti-rabbit lgG, or Alexa Fluor®594 donkey anti-rabbit lgG, or Alexa Fluor®594 donkey anti-mouse lgG, life technologies) for 30 min, and subsequently incubated with DAPI. The images were observed and collected under fluorescence microscope. Three random fields in 200× were selected for quantification. ImageJ software was used to analyze the fluorescence intensity of CD44v6, MSI2 and Notch1. The experiments were repeated independently three times.

### Cell culture and reagents

Human hepatic L02 cell line, HCC cell lines MHCC-97 h, MHCC-97 L were provided by the Cell Bank of the Chinese Academy of Sciences (Shanghai, China), SNU-398, PLC/PRF/5 were obtained from the American Type Culture Collection (ATCC, Manassas, VA, USA), HLE, Huh-7 were obtained from the Japanese Cancer Research Bank (Tokyo, Japan). SNU-398 were cultured in RPMI 1640 Medium (GIBCO), the rest of the cell lines were cultured in Dulbecco’s Modified Eagle’s Medium (DMEM; GIBCO, Grand Island, NY, USA). All medium was supplemented with 10% fetal bovine serum (FBS; GIBCO) and 1% penicillin/streptomycin (Invitrogen). Cells were cultured in a humidified atmosphere of 5% CO_2_ at 37 °C. The Notch signaling pathway inhibitor RO4929097 was purchased from MedChemExpress (MCE).

### Flow cytometry analysis

Cultured cells (5 × 10^5^ cells) were centrifuged. The cell pellets were suspended in FACS buffer (PBS containing 0.5% fetal bovine serum (FBS)) and then labeled with PE-conjugated human CD44v6 antibody (Clone 2F10, R&D Systems, #FAB3660P) at 4 °C for 30 min. Cells were washed twice, re-suspended in FACS buffer and were analyzed with the FACS Calibur machine using CellQuest software (BD Biosciences).

### Magnetic bead cell sorting

CD44v6 expression were examined by cytometry (FACS) analysis in human hepatic L02 cells (0.64%) and HCC cell lines (HepG2 (0.86%), MHCC-97 L (1.96%), SMMC-7721 (3.75%), HLE (6.82%), Huh-7 (14.5%), MHCC-97 h (16.6%) and SNU-398 (46.1%)) (Additional file 1: Figure S2A and S2B). Then we enriched CD44v6+ and CD44v6- populations from MHCC-97 h and SNU-398 cell lines by magnetic bead cell sorting and identified the sorting efficiency by FACS (Additional file 1: Figure S2C). CD44v6+ LCSCs and CD44v6- cells were isolated by EasySep™ Human PE Positive Selection Kit (STEMCELL Catalog #18551 and 17664). All the procedures were performed based on the manufacturer’s protocol. Briefly, prepare single cells in the recommended medium (PBS containing 2% fetal bovine serum (FBS) and 1 mM EDTA) with a concentration of 2 × 10^8^ cells/ ml. Then incubate 15 min at room temperature (RT) after adding FcR blocker and PE-conjugated CD44v6 antibody (FAB3660P, R&D Systems (RD)), RT for 15 min after adding selection cocktail and RT for 10 min after adding magnetic particles. Place the tube into the magnet (EasySep™, Catalog #18000) to harvest the CD44v6+/CD44v6- cells. Quality of sorting was monitored by flow cytometry.

### Sphere formation assay

For spheroid culture, cells were suspended into single-cell in serum-free DMEM/F12 medium (cat#12400–024, GIBCO, Grand Island, NY) with 100 IU/ml penicillin, 100 μg/ml streptomycin, 20 ng/ml human recombinant epidermal growth factor (EGF, cat#PHG0311; GIBCO), 10 ng/ml human recombinant basic fibroblast growth factor (bFGF, cat#PHG0266;GIBCO), 2% B27 supplement (cat#17504–044; GIBCO, Grand Island, NY), 1% N-2 supplement (cat#17502–048; GIBCO, Carlsbad, CA, USA), and 1% methyl cellulose (cat#M0262; Sigma-Aldrich). Then plated in ultra-low attachment 24-well plates (Corning, NY, USA) at a density of 5 × 10^3^ cells/ml. Spheres more than 100 μm in diameter were counted microscopically. The experiments were repeated independently three times.

### Xenograft tumor model

All experiments with mice approved by Tongji Hospital Institutional Review Board (IRB ID: TJ-A20161211). Four-weeks-old male NOD/SCID mice were obtained from Beijing Huafukang Biotechnology company and maintained in pathogen-free conditions. Mouse orthotopic liver xenograft tumor model and subcutaneous xenograft tumor model two types of mouse models were used. In subcutaneous xenograft tumor model, 1 × 10^5^, 1 × 10^4^, 1 × 10^3^ CD44v6+ cells or CD44v6- cells were injected subcutaneously. In mouse orthotopic liver xenograft tumor model, 1 × 10^5^ CD44v6+ cells or CD44v6- cells were injected into the left lobes of the liver. For the orthotopic liver xenograft tumor model, animals were sacrificed 4–5 weeks after implantation for the mice injected in the liver. Bioluminescence was measured 5 minutes after tail intravenous injection administration 100ul of potassium D-luciferin salt (30 mg/mL) dissolved in PBS (per animal). For the mice injected subcutaneously, mice were sacrificed 4 weeks after injection and examined for the growth of subcutaneous tumors. Tumor growth was followed with a caliper, and tumor volume = xy^2^/2, x is the longest and y is the shortest of two perpendicular diameters.

### Lentivirus

Lentiviral particles expressed MSI2 shRNA, MSI2, Notch1 shRNA, LFNG shRNA or LFNG were obtained from Genechem, Shanghai, were used to down-regulate or up-regulate MSI2, Notch1 and LFNG in sorted HCC cells. The sequence for lentivirus-based RNAi targeted MSI2 was listed as follow: MSI2 shRNA1, 5′-ATAGCCTTAGAGACTATTT-3′, MSI2 shRNA2, 5′- AGCAAGTGTAGATAAAGTA-3′ and control sequence for nonspecific and off-target effects was: 5′-TTCTCCGAACGTGTCACGT-3′. The sequence for lentivirus-based RNAi targeted Notch1 was listed as follow: 5′-GGAGCATGTGTAACATCAA-3′. And the sequence for lentivirus-based RNAi targeted LFNG was listed as follow: LFNG shRNA1 5′-GAGCTACGGTATGTTTGAA-3′. LFNG shRNA2 5′- ACTGCACCATCGGCTACAT-3′, LFNG shRNA3 GCAACGTGGTCATCACAAA. LFNG shRNA1 was the most effective shRNA and was used in the subsequent experiments (Additional file 3: Figure S5E).

### RNA and reverse transcription-PCR

The cDNA were created according to the manufacturer’s protocol (Takara, PrimeScript RT Master Mix). Quantitative PCR was performed on StepOne Real-Time System (Bio-rad) using SYBR Premix Ex TaqTM (Takara, DRR081A) according to the manufacturer’s protocol. Gene expression was normalized to β-actin mRNA content for human genes, and expressed relatively to the control condition of each experiment. The relative expression of each target gene was determined from replicate samples using the 2^-ΔΔCt^ (Ct, cycle threshold). Primer sequences were provided in Additional file 4: Table S3.

### Notch RT^2^ PCR Array

CD44v6+ LCSCs were transfected with MSI2 shRNA1 lentivirus to down-regulate MSI2. Magnetic bead cell sorting experiments were conducted to enrich CD44v6+ SNU-398 cells. The sorted CD44v6+ cells were divided into two groups and transfected with MSI2 shRNA1 lentivirus and control lentivirus, which were named as MSI2 shRNA1–1 group and NC1 group. Cell sorting experiment and lentivirus transfection experiment were repeated twice, cells obtained from the repeated experiments were named as MSI2 shRNA1–2 group and NC2 group. A Notch RT^2^ PCR Array (QIAGEN, PAHS-059Z RT^2^ PCR Array) which contain 84 Notch pathway-focused genes as well as five housekeeping genes (ACTB, B2M, GAPDH, HPRT1, RPLP0) was used to compare the mRNA expression between MSI2 shRNA1 cells and control CD44v6+ LCSCs. Total RNA was extracted using RNAiso Plus (Takara, Japan) and quantified by a Nanodrop 2000 (ThermoFisher Scientific). The cDNA synthesis was performed according to the manufacturer’s instructions (Takara, PrimeScript RT Master Mix). The cDNA was used on the real-time RT^2^ Profiler PCR Array (QIAGEN, Cat. no. PAHS-059Z) in combination with RT^2^ SYBR® Green qPCR Mastermix (Cat. no. 330529). Data were extracted using the following criteria: *P*-value ≤0.05. The array included controls to assess cDNA quality and DNA contamination.

### Western blot

Western blot analysis was performed as described previously [26]. Primary antibodies include Notch1 (CST, cat#3608), cleaved Notch1 (CST, Val1744, D3B8, cat#4147), Hes1 (CST, cat#11988), Hey1 (Abcam, ab154077), Nanog (CST, cat#4903), Sox2 (CST, cat#3579), Oct4 (CST, cat#2750), MSI2 (Abcam, ab76148), LFNG (CST, cat #66472), Numb (CST, cat #2756). Anti-β-actin (Abcam, ab8226) was used as an internal control. Immune complexes were visualized using the Beyo ECL Plus.

### Transwell migration and invasion assay

Cell migration was analyzed using Transwell chambers (8-μm pore size; Millipore, Billerica, MA, USA), and cell invasion was analyzed using this Transwell chambers with a Matrigel (BD Biosciences, San Jose, CA, USA) matrix. Cells were plated with FBS free culture medium in the upper chamber and 10% FBS culture medium as a chemoattractant. After 28 h (Migration) or 32 h (Invasion) of incubation, the low surface of the plates containing cells were washed with PBS, fixed in methanol, stained with a 4 g/L crystal violet solution and imaged. Photographs of three randomly selected fields of the fixed cells were captured and cells were counted. The experiments were repeated independently three times.

### Colony formation assay

Cells were seeded at a density of 1000 cells per well in the 6-well plates. After 2 weeks’ incubation at 37 °C, clones were fixed by 4% methanol and stained with a 4 g/L crystal violet solution. Clone (> 50 cells) numbers were counted under the microscope. The experiments were repeated independently three times.

### CCK8 toxic assay

The sensitivity of cells to Sorafenib was measured by CCK8 assay. Cells were seeded in 96-well plates at a density of 1000 cells per well and then treated with various concentrations (2.5 μM, 5 μM, 10 μM or 20 μM) of sorafenib (Sigma-Aldrich) after the cells were attached. Then incubated for 24 h, replaced fresh culture medium and added cell counting kit-8 (CCK8, promoter, China) to each well according to the manufacturer’s protocol, incubated at 37 °C for 2 h. Absorbance was measured at 450 nm using a microplate reader (Thermo Scientific).

### Co-immunoprecipitation

Co-immunoprecipitation was performed according to the manufacturer’s protocol. Briefly, 5 × 10^7^ SNU-398 cells were harvested and lysed with RIPA lysis buffer containing protease inhibitors cocktail. Cell extracts were incubated with Protein A/G PLUS-Agarose (Santa Cruz) and an appropriate control IgG. Then incubated with MSI2 (Abcam, ab114083) or LFNG (CST, cat#66472) primary antibody after centrifugation. Subsequently, the cell lysates were incubated with Protein A/G PLUS-Agarose (Santa Cruz). Then the protein A/G PLUS-Agarose was collected, washed and boiled, samples were immunoblotted with anti-MSI2 (ORIGENE, cat#TA506196S) or anti-LFNG (ABGENT, cat#AP9524c-400 1) antibodies.

### RNA immunoprecipitation (RIP) assay

RIP assays were performed using the Magna RIP™ RNA-Binding Protein Immunoprecipitation Kit (no.17–700; Millipore, USA) according to the manufacturer’s protocol. Briefly, 3 × 10^7^ SNU-398 cells were harvested and lysed with RIP lysis buffer provided in the kit. Five microgram anti-MSI2 (ab114083, Abcam) antibodies or anti-rabbit IgG antibodies were incubated with magnetic beads and used to precipitate MSI2-RNA complexes. Then the complexes were washed and treated with proteinase K. RNA were extracted using the phenol/chloroform method, and the retrieved RNA was subjected to agarose electrophoresis analysis using LFNG-specific primers. Total RNA (input controls) and normal rabbit IgG controls were assayed simultaneously to confirm that the detected signals were from the RNA specifically binding to MSI2. SNRNP70 served as positive controls, while U1 served as negative controls, respectively (Additional file 3: Figure S6). LFNG: 138 bp, Forward Primer GTCAGCGAGAACAAGGTGC; Reverse Primer GATCCGCTCAGCCGTATTCAT.

### Statistical analysis

Results were analyzed using the GraphPad Prism 6.0 statistical software. For comparisons between two groups, parametric Student t-test was used. For comparisons between more than two groups, parametric One-Way analysis of variance (ANOVA) test followed by a posteriori Bonferroni test was used. Statistical analysis was conducted using SPSS statistical software (SPSS Inc., Chicago, IL, USA), version 22.0. Survival curves were analyzed using the Kaplan–Meier method, and significance was assessed by the log-rank test. In all assays, *p* ≤ 0.05 was considered as statistical significance.

## Results

### MSI2 was positively correlated with CD44v6 expression and predicted poor prognosis in patients with HCC

**Fig. 1:**
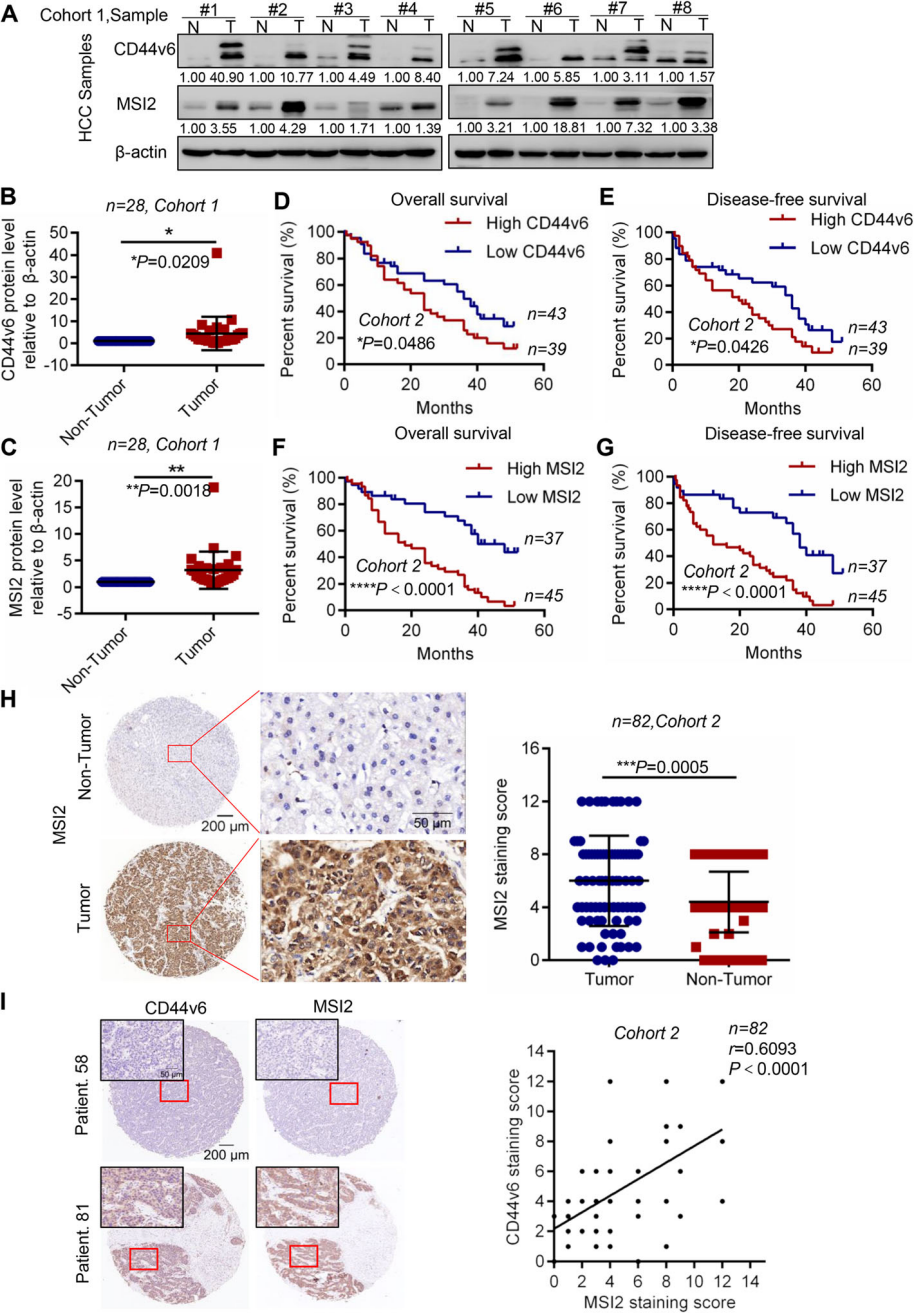
MSI2 was closely related to CD44v6 and predicted poor prognosis. **a** Western blot analysis of CD44v6 and MSI2 protein levels in HCC tissues and adjacent non-tumor tissues selected randomly. β-actin was used as a normalized control. **b** Analysis of CD44v6 protein levels relative to β-actin in 28 pairs of HCC tissues and adjacent non-tumor tissues (*n* = 28, *p = 0.0179, *t* test). **c** Analysis of MSI2 protein levels relative to β-actin in 28 pairs of HCC tissues and adjacent non-tumor tissues (*n* = 28, **p = 0.0012, *t* test). **d** and **e** Kaplan–Meier survival analysis of overall survival and disease-free survival were compared according to the expression levels of CD44v6 in HCC tissues. Patients with high CD44v6 expression had shorter overall survival (**d**, median survival = 24 months Vs. 36 months, log-rank test, *n* = 82, *p = 0.0486) and disease-free survival (**e**, median survival = 20 months Vs. 36 months, log-rank test, *n* = 82, **p = 0.0426). **f** and **g** Kaplan–Meier survival analysis of overall survival and disease-free survival were compared according to the expression levels of MSI2 in HCC tissues. Patients with high MSI2 expression had shorter overall survival (**f**, median survival = 18 months Vs. 40 months, log-rank test, *n* = 82, ****p<0.0001) and disease-free survival (**g**, median survival = 12 months Vs. 38 months, log-rank test, *n* = 82, ****p<0.0001). **h** Representative images of IHC staining of MSI2 in tumor and adjacent non-tumor tissues. And analysis of MSI2 expression in tumor and adjacent non-tumor tissues by paired *t* test. Scale bars: 200 μm and 50 μm. **i** The expression of MSI2 and CD44v6 in tumor tissues from the same HCC patient were analyzed by IHC staining and found that MSI2 was positively correlated with CD44v6 (*n* = 82, *r* = 0.6093, ****p<0.0001, Pearson’s correlation). Scale bars: 200 μm and 50 μm

Firstly, expression of CD44v6 and MSI2 at the protein level was measured by western blot analysis with 28 paired HCC samples in Cohort 1. The results showed that the expression of CD44v6 and MSI2 in HCC tissues was significantly higher than those in peri-tumor tissues (Fig. 1a, b, c and Additional file 1: Figure S1A). Additionally, to evaluate the possible association between MSI2 and CD44v6 expression and the prognostic role of CD44v6 and MSI2 in human HCC tissues, we detected their expressions in a tissue microarray with 82 paired tissues from patients with HCC by Immunohistochemistry (tumor tissues versus adjacent non-tumor tissues, Cohort 2), and analyzed their relationship. The results showed that HCC patients with high CD44v6 expression had shorter overall survival (median survival = 24 months vs. 36 months) and disease-free survival (median survival = 20 months vs. 36 months) than that of patients with low CD44v6 expression (Fig. 1d and e, *P = 0.0486 and * *P* = 0.0426, respectively, log-rank test). We examined the correlation of CD44v6 expression with clinicopathologic features and the results showed that CD44v6 expression was positively correlated with advanced stage (Table 1, * *P*<0.05; χ^2^ test). Furthermore, we analyzed the prognostic significance of MSI2, which shown that HCC patients with high MSI2 expression had shorter overall survival (median survival = 18 months vs. 40 months) and disease-free survival (median survival = 12 months vs. 38 months) than patients with low MSI2 expression (Fig. 1f and g, ****P<0.0001 and ****P<0.0001, respectively, log-rank test). Moreover, consistent with the results of Cohort 1, we found that the expression of MSI2 was dramatically higher in HCC tumors compared to adjacent non-tumor tissues (Fig. 1h, *n* = 82, *** *P* = 0.0005, t test), Representative cases of immunohistochemical staining of MSI2 and CD44v6 were shown in Fig. 1h, Additional file 1: Figure S1B and S1C. Moreover, clinicopathologic statistics of MSI2 in HCC patients demonstrated that high expression of MSI2 was significantly correlated with less tumor differentiation (Table 1; ** *P*<0.01; χ^2^ test). Importantly, we observed that MSI2 expression was positively correlated with CD44v6 expression in HCC patients (Fig. 1i; *n* = 82, *r* = 0.6093, **** *P*<0.0001, Bilateral, Pearson’s correlation). In summary, these results indicated that higher expression of MSI2 and CD44v6 predicted poor prognosis in HCC patients. The expression of MSI2 was positively correlated with CD44v6.

**Table 1:** Correlation of the expression of CD44v6 and MSI2 with clinical-pathological variables in HCC patients

Clinical-Pathological Variables	Low CD44v6 (*n* = 43)	High CD44v6(*n* = 39)	*P*-value	Low MSI2(*n* = 37)	High MSI2(*n* = 45)	*P*-value
Age(years)						
Young(≤median,48)	27	20	*P* = 0.293	22	25	*P* = 0.722
Old(>median,48)	16	19		15	20	
AFP(ng/ml)						
Low(≤20)	8	13	*P* = 0.127	11	10	*P* = 0.438
High(>20)	35	26		26	35	
HBsAg						
Negative	9	3	*P* = 0.090	7	5	*P* = 0.320
Positive	34	36		30	40	
GGT(U/I)						
Low(≤50)	10	17	*P* = 0.050	10	17	*P* = 0.303
High(>50)	33	22		27	28	
Tumor Differentiation						
Well differentiated	10	7	*P* = 0.554	13	4	**P = 0.004
Moderately to poorly differentiated	33	32		24	41	
Vascular Invasion						
Absence	34	34	*P* = 0.330	30	38	*P* = 0.687
Presence	9	5		7	7	
Tumor Size						
Small(≤5 cm)	21	13	*P* = 0.155	11	23	*P* = 0.051
Large(>5 cm)	22	26		26	22	
Lymph Node Metastasis						
Absence	40	34	*P* = 0.373	32	42	*P* = 0.298
Presence	3	5		5	3	
TNM stage^a^						
Early stage (I—II)	21	10	*P= 0.031	11	20	*P* = 0.171
Advanced stage (III—IV)	22	29		26	25	

^a^ AJCC/UICC T staging system

*P < 0.05, **P<0.01,Significant difference, (χ2 test and Fisher’s exact test)

### CD44v6+ cells possessed the characteristics of liver cancer stem cells

**Fig. 2:**
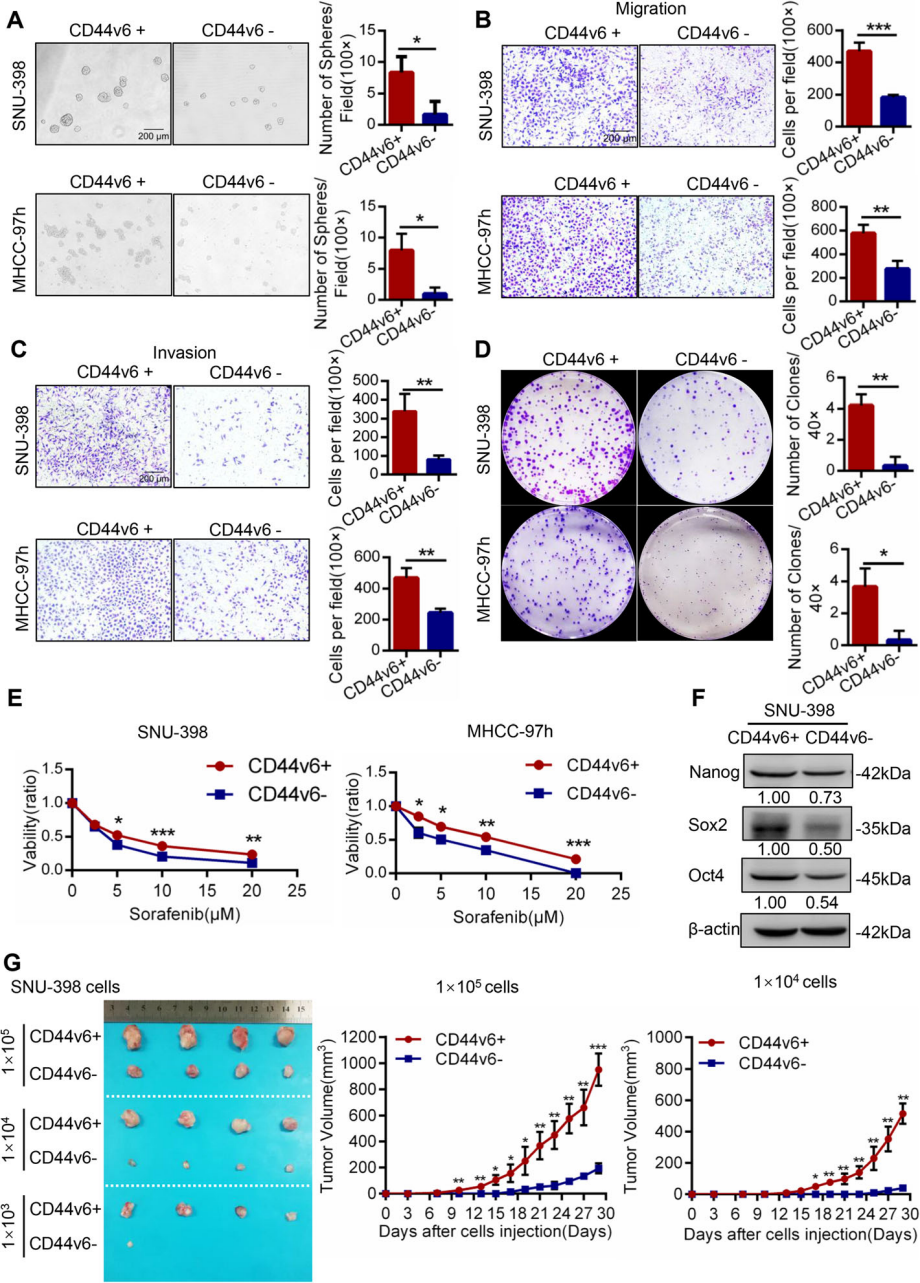
CD44v6+ HCC cells possessed characteristics of cancer stem cells. **a** Representative images of spheres and histogram analysis in indicated cells. CD44v6+ SNU-398 cells and MHCC-97 h cells processed enhanced self-renewal property than CD44v6- cells. Scale bar, 200 μm. **b** and **c** Representative images of transwell migration and invasion in indicated cells. Transwell migration and invasion assays showed that CD44v6+ SNU-398 cells and MHCC-97 h cells displayed higher migratory and invasion capacity than CD44v6- cells. Scale bar, 200 μm. **d** Representative images of colony formation assays in indicated cells. Colony formation assays demonstrated that CD44v6+ SNU-398 cells and MHCC-97 h cells exhibited higher proliferation and colony formation ability. **e** CD44v6+ and CD44v6- SNU-398 cells and MHCC-97 h cells were treated with Sorafenib for 24 h and evaluated by CCK8 toxic assay. It showed that CD44v6+ cells were more resistant to Sorafenib than CD44v6- cells. **f** The expression of cancer stemness-related genes, including Nanog, Oct4 and Sox2 in CD44v6+ and CD44v6- SNU-398 cells. β-actin was used as a normalized control. It showed that elevated stemness-related genes expressed in CD44v6+ SNU-398 cells than CD44v6- cells. **g** Efficiency of tumor formation of CD44v6+ cells and CD44v6- cells isolated from SNU-398 cell line. Number of injected cells: 1 × 10^5^, 1 × 10^4^, 1 × 10^3^. *n* = 12. For statistical analysis, * *p* < 0.05, **p < 0.01 and ***p < 0.001, t test

CD44v6 expression was examined by flow cytometry (FACS) analysis in human hepatic L02 cells (0.64%) and HCC cell lines (HepG2 (0.86%), MHCC-97 L (1.96%), SMMC-7721 (3.75%), HLE (6.82%), Huh-7 (14.5%), MHCC-97 h (16.6%) and SNU-398 (46.1%)) (Additional file 1: Figure S2A and S2B). It showed that the expression of CD44v6 in normal human hepatic cell line was lower than HCC cell lines. Then we isolated CD44v6+ and CD44v6- cells from MHCC-97 h and SNU-398 cell lines by magnetic bead cell sorting and identified the sorting efficiency by FACS (Additional file 1: Figure S2C). Stemness properties of CD44v6+ HCC cells were tested by a series of in vitro and in vivo experiments. Sphere formation assays showed that CD44v6+ SNU-398 cells and MHCC-97 h cells could form larger and more spheres than CD44v6- cells, indicating that CD44v6+ cells possessed enhanced self-renewal ability than CD44v6- cells (Fig. 2a). Transwell migration and invasion assays showed that CD44v6+ SNU-398 cells and MHCC-97 h cells displayed higher migratory and invasion capacity than CD44v6- cells (Fig. 2b and c). Moreover, colony formation assays demonstrated that CD44v6+ SNU-398 cells and MHCC-97 h cells exhibited higher proliferation and colony formation ability (Fig. 2d). Furthermore, CD44v6+ SNU-398 cells and MHCC-97 h cells were treated with various concentration of Sorafenib for 24 h, we found that CD44v6+ cells were more resistant to Sorafenib than CD44v6- cells (Fig. 2e). Western blot showed that CD44v6+ SNU-398 cells expressed more stemness-related genes (Nanog, Oct4, and Sox2) than CD44v6- cells (Fig. 2f).

In vivo, the tumor-initiating capacity of CD44v6+ cells was evaluated with subcutaneous xenograft tumor model in immunodeficiency mice. 1 × 10^5^, 1 × 10^4^, 1 × 10^3^ CD44v6+ cells and CD44v6- cells purified from SNU-398 cells were implanted subcutaneously into mice. The results showed that 1 × 10^3^ CD44v6- cells could form tumor in only 1 mouse, whereas 1 × 10^3^ CD44v6+ cells could form tumor in all mice over the same period (1/4 vs. 4/4 in 1 × 10^3^ group, Additional file 4: Table S1). Moreover, the volume of tumors derived from CD44v6+ cells were bigger than those from CD44v6- cells (Fig. 2g). Therefore, CD44v6+ cells possessed significantly higher tumorigenic capacity than CD44v6- cells. Taken together, these data demonstrated that CD44v6+ cells possessed a higher capacity of self-renewal, migration, invasion, resistance to Sorafenib, tumorigenic ability and expressed more stemness-related genes than CD44v6- cells.

### MSI2-maintained CD44v6+ HCC cell self-renewal, metastatic capacity, and tumorigenic ability in vitro and in vivo

**Fig. 3:**
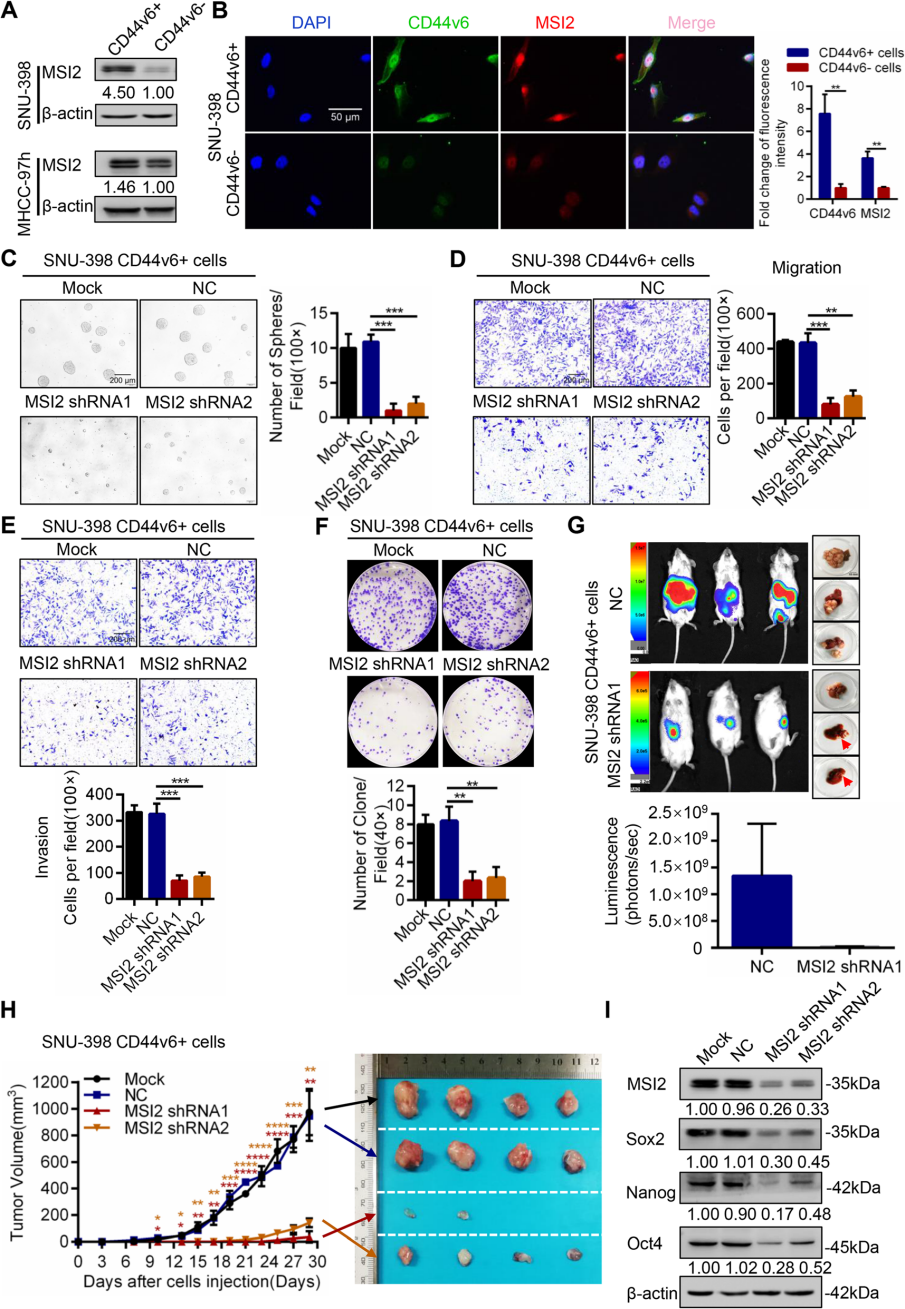
Knockdown of MSI2 significantly attenuated the stemness properties of CD44v6+ LCSC. **a** MSI2 expression levels were tested in CD44v6+ HCC cells and CD44v6- HCC cells by western blot in SNU-398 and MHCC-97 h cell lines. **b** Immunofluorescence images of CD44v6+ SNU-398 cells and CD44v6- SNU-398 cells for localization of MSI2 (red) and CD44v6 (green). Histogram analysis of the relative fluorescence intensity of CD44v6 and MSI2 in CD44v6+ cells and CD44v6- cells. Scale bar, 50 μm. **c** Representative images of spheres and histogram analysis in indicated cells. The inhibition of MSI2 decreased self-renewal property in vitro in CD44v6+ LCSCs, Scale bar, 200 μm. **d** and **e** Transwell migration and invasion assays showed that knockdown of MSI2 decreased the migration and invasion of CD44v6+ cells. Scale bar, 200 μm. **f** Colony formation assays showed that the ability of cell proliferation and colony formation of CD44v6+ cells was inhibited when MSI2 was down regulated. **g** 1 × 10^5^ of MSI2 shRNA1 cells and the corresponding controls were injected into the left lobes of liver. Bioluminescence signals from MSI2 shRNA1 groups were weaker than that from corresponding control groups. Red arrows indicated the site of tumor formation. **h** Efficiency of tumor formation of MSI2 shRNA cells and corresponding control cells. Number of injected cells: 1 × 10^5^. *n* = 8. Black arrow means Mock group, blue arrow means NC group, red arrow means MSI2 shRNA1 group, and orange arrow means MSI2 shRNA2 group. **i** The expression of cancer stemness-related genes, including Nanog, Oct4 and Sox2 in MSI2 shRNA cells compared with corresponding control. β-actin was used as a normalized control. It showed the expression of stemness-related genes were decreased when MSI2 was knockdown in CD44v6+ LCSCs. For statistical analysis, *p < 0.05, **p < 0.01, ***p < 0.001 and ****p < 0.0001, t test

Recent studies demonstrated that MSI2 contributed to the phenotype of CSCs [11, 28]. To explore the role of MSI2 in the maintenance of CD44v6+ LCSCs stemness properties, we analyzed the expression of MSI2 in CD44v6+ and CD44v6- cells purified from MHCC-97 h and SNU-398 cell lines. Western blot showed that MSI2 expression was higher in CD44v6+ cells than CD44v6- cells in MHCC-97 h and SNU-398 human HCC cell lines (Fig. 3a). By using immunofluorescence, we found more MSI2 (red) expression in CD44v6+ cells than CD44v6- cells. MSI2 (red) and CD44v6 (green) co-localized in the nucleus and cytoplasm of SNU-398 cell line (Fig. 3b). These results indicated that MSI2 was enriched in CD44v6+ LCSCs.

We further investigated the role of MSI2 in maintaining stemness properties in CD44v6+ LCSCs by downregulating MSI2 (using MSI2 shRNA) in CD44v6+ cells or overexpressing MSI2 (using Lentiviral MSI2) in CD44v6- cells. Sphere formation assays showed that down-regulation of MSI2 expression significantly decreased the self-renewal ability of CD44v6+ cells (Fig. 3c). Whereas overexpression of MSI2 significantly increased the self-renewal ability in CD44v6- cells (Additional file 2: Figure S3A). Transwell migration and invasion assays showed that knockdown of MSI2 decreased the migration and invasion capacity of CD44v6+ cells (Fig. 3d and e). Enhanced MSI2 expression increased the migration and invasion capacity of CD44v6- cells (Additional file 2: Figure S3B and S3C). Moreover, colony formation assays demonstrated that MSI2 knockdown in CD44v6+ cells significantly inhibited proliferation and colony formation of CD44v6+ cells (Fig. 3f). MSI2 overexpression increased the colony formation of CD44v6- cells (Additional file 2: Figure S3D). Furthermore, down-regulation of MSI2 in CD44v6+ cells significantly decreased resistance to Sorafenib compared with the control (Additional file 2: Figure S3G). In vivo, the effect of MSI2 knockdown on CD44v6+ cells was examined in a mouse orthotopic liver xenograft tumor model and subcutaneous xenograft tumor model in NOD/SCID mice. The interference efficiency of MSI2 shRNA1 was higher than that of MSI2 shRNA 2 and was used in the mouse orthotopic liver xenograft tumor model (Fig. 3i). The results showed that luminescence intensity from MSI2 shRNA1 cells was weaker than that derived from scrambled cells (Fig. 3g, mean luminescence intensity: 8.06e+ 6 vs. 1.34e+ 9, 1 × 10^5^ CD44v6+ cells). The volume of tumors derived from MSI2 shRNA cells was smaller than that derived from scrambled cells in subcutaneous xenograft tumor model (Fig. 3h, 1 × 10^5^ CD44v6+ cells). In line with these results, when the MSI2 was overexpressed in CD44v6- cells with Lv MSI2, the luminescence intensity from Lv MSI2 cells was stronger than that derived from scrambled shRNA-treated cells (Additional file 2: Figure S3E, mean luminescence intensity: 1.65e+ 9 vs. 6.06e+ 8; 1 × 10^5^ CD44v6- cells).

Finally, we found that down-regulation of MSI2 decreased the expression of stemness-related genes (Nanog, Oct4, and Sox2) in CD44v6+ HCC cells (Fig. 3i and Additional file 2: Figure S3H). Whereas up-regulation of MSI2 in CD44v6- HCC cells increased the expression of stemness-related genes (Additional file 2: Figure S3F). Collectively, these results indicated that MSI2 was essential for maintaining the stemness properties for CD44v6+ LCSCs.

### Inhibition of Notch1 pathway attenuated CD44v6+ LCSCs’ self-renewal, invasion and tumorigenic capacity in vitro and in vivo

**Fig. 4:**
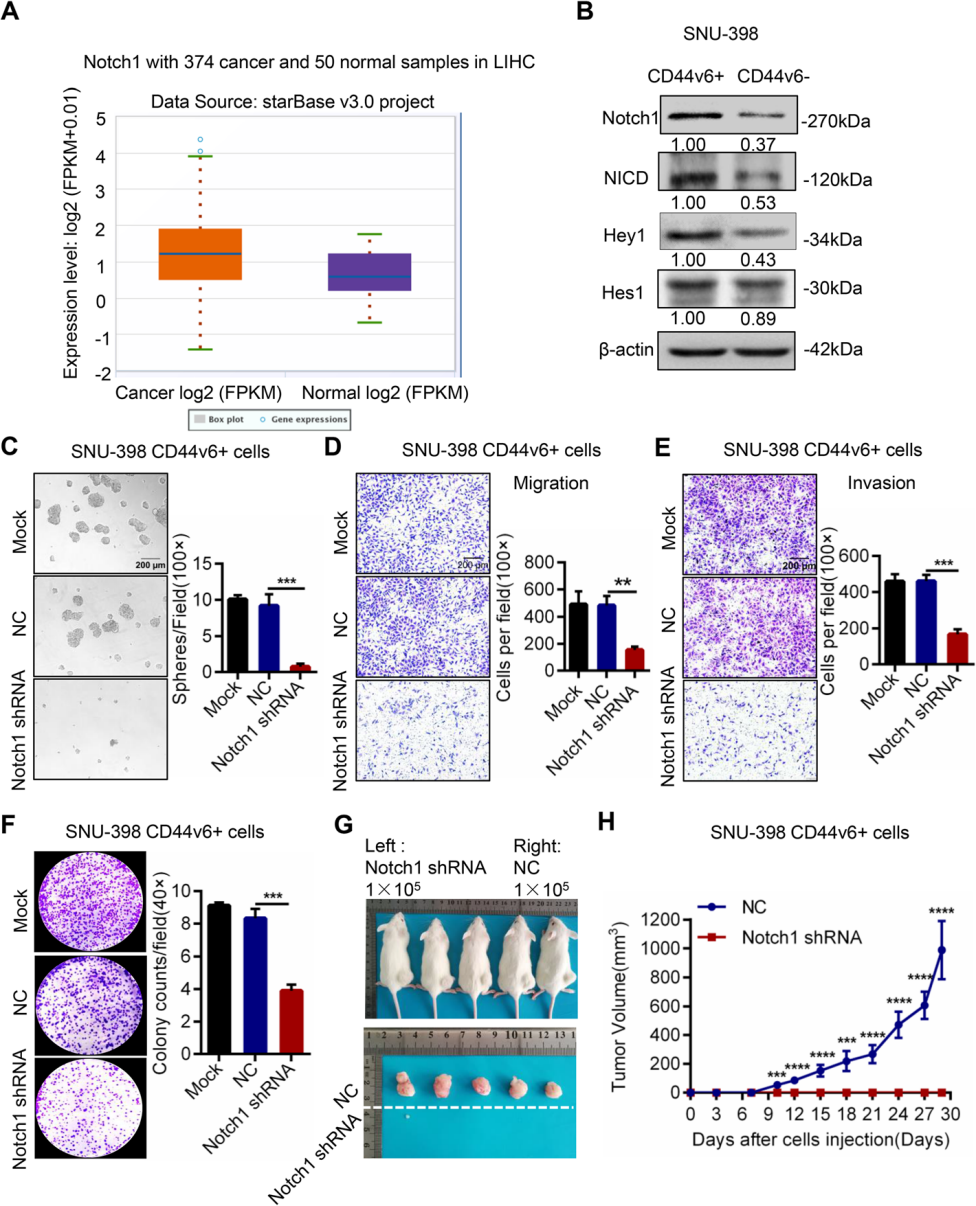
The inhibition of Notch1 signaling pathway attenuated the stemness properties of CD44v6+ LCSCs. **a** External dataset from starBase v3.0 project with 374 HCC samples and 50 normal samples was used to analyze the expression of Notch1. The result showed that Notch1 was higher in HCC patients’ samples than that in normal samples (1.77 fold, FDR = 0.0017). **b** The core components of Notch1 signaling including Notch1 receptor, cleaved Notch1 (NICD), Hey1 and Hes1 were tested in CD44v6+ LCSCs and CD44v6- HCC cells by western blot in SNU-398 cell lines. Western blot showed that CD44v6+ SNU-398 cells expressed more Notch1 signaling pathway key factors. β-actin was used as a normalized control. **c** Representative images of spheres and histogram analysis in indicated cells. The inhibition of Notch1 decreased self-renewal property in vitro in CD44v6+ LCSCs, Scale bar, 200 μm. **d** and **e** Transwell migration and invasion assay showed that knockdown Notch1 decreased the migration and invasion capacity of CD44v6+ cells. Scale bar, 200 μm. **f** Colony formation assays showed that the ability of cell proliferation and colony formation of CD44v6+ cells was inhibited when Notch1 was down regulated. **g** and **h** Efficiency of tumor formation of Notch1 shRNA cells and the corresponding controls. Right flanks of mice were injected with control CD44v6+ cells while left flanks were injected with Notch1 shRNA cells. Number of injected cells: 1 × 10^5^. *n* = 5. Data are expressed as mean ± SD (error bars). ** *p* < 0.01, ***p < 0.001 and ****p < 0.0001, t test

Our previous studies have demonstrated that the Notch signaling pathway plays a crucial role in promoting the stemness properties of LCSCs, and that inhibition of Notch signaling attenuates the stemness of LCSCs [25, 26]. Consistent with our previous studies, we found that the expression of Notch1 was higher in the tumors from HCC patients than in livers of healthy individuals (Fig. 4a, 1.77 fold, FDR = 0.0017) using the external dataset from starBase v3.0 project which consists of 374 HCC samples and 50 normal samples.

To examine whether Notch1 signaling was activated in CD44v6+ LCSCs, we measured the expression of the key components in Notch1 signaling in CD44v6+ and CD44v6- cells. Elevated expression of Notch1, NICD (cleaved Notch1), and the downstream target genes Hey1 and Hes1 were detected in CD44v6+ cells compared to CD44v6- cells (Fig. 4b). To further investigate the function of Notch1 signaling pathway in CD44v6+ LCSCs, we repressed Notch1 expression genetically (Notch1 shRNA) or its activity pharmacologically (γ-secretase inhibitor RO4929097) (Additional file 2: Figure S4A and B) and then evaluated the stemness properties in CD44v6+ LCSCs. Notably, deletion of Notch1 remarkably decreased the number of spheroids formed by CD44v6+ HCC cells (Fig. 4c). Transwell migration and invasion assays demonstrated that Notch1 knockdown also significantly decreased the migration and invasion capacity of CD44v6+ HCC cells (Fig. 4d and e). Moreover, colony formation assays showed that silencing of Notch1 prevented colony formation by CD44v6+ HCC cells (Fig. 4f). Furthermore, we assessed the impact of the Notch1 pathway on tumorigenic capacity using subcutaneous xenografts model in NOD/SCID mice. The results revealed that silencing Notch1 expression inhibited CD44v6+ LCSCs tumorigenic capacity (Fig. 4g and h, 1 × 10^5^ cells).

Consistently, the γ-secretase inhibitor RO4929097 suppressed the stemness properties of CD44v6+ LCSCs (Additional file 2: Figure S4C-F). RO4929097 treatment decreased the expression of stemness-related genes in CD44v6+ LCSCs (Additional file 2: Figure S4G). In conclusion, the inhibition of Notch1 signaling diminished the stemness properties of CD44v6+ LCSCs, indicating that the Notch1 pathway played an important role in the maintenance of CD44v6+ LCSCs.

### MSI2 maintained the stemness properties of CD44v6+ LCSCs through activating Notch1 signaling pathway

**Fig. 5:**
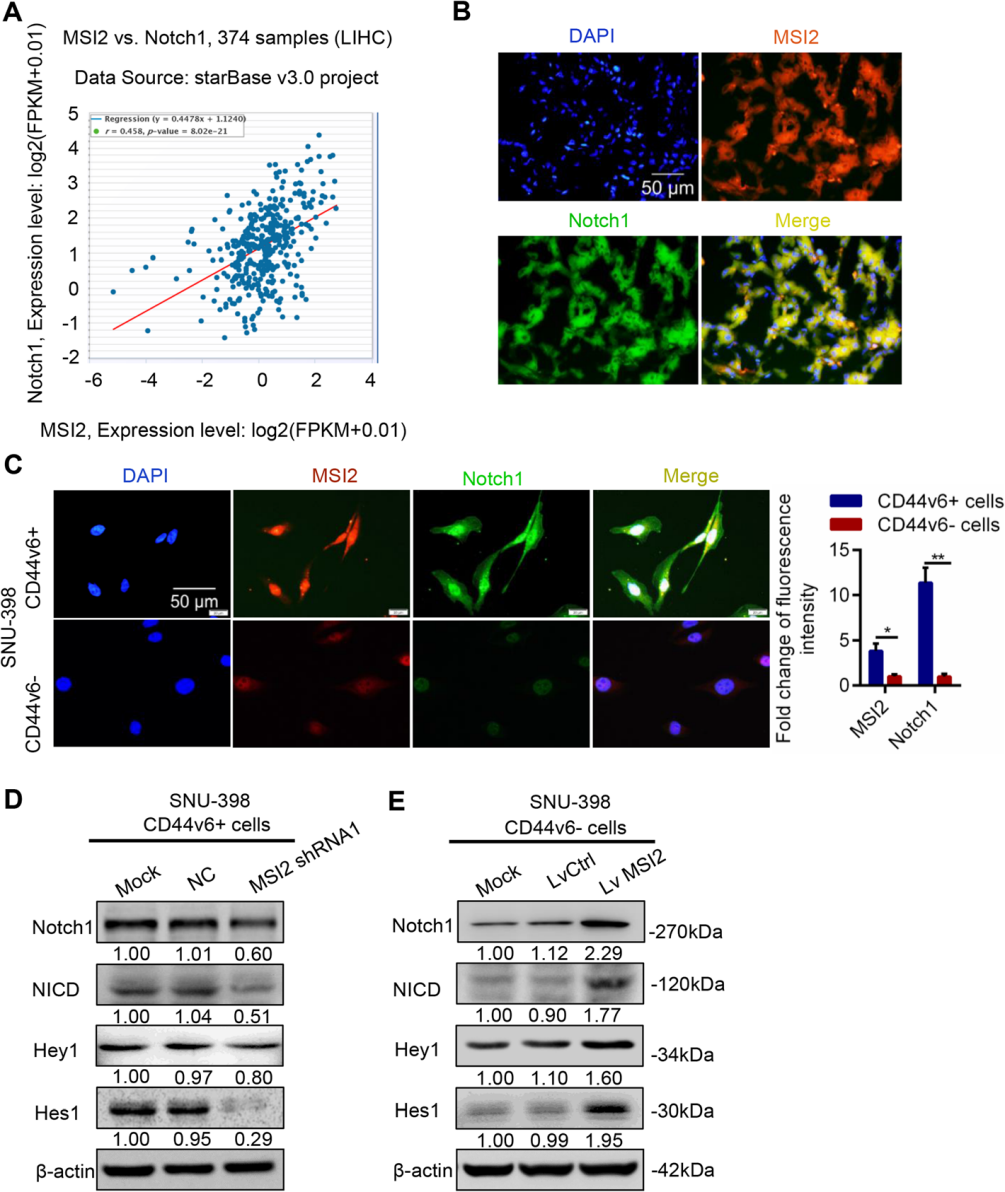
MSI2 maintained the stemness properties of CD44v6+ LCSCs via activating Notch1 signaling pathway. **a** External dataset from starBase v3.0 project with 374 HCC samples and 50 normal samples was used to analyze the correlation of MSI2 and Notch1. The results showed that MSI2 was positively related to Notch1 in clinical-pathology (*r* = 0.458, *p* = 8.02e-21). **b** Immunofluorescence images of MSI2 (red) and Notch1 (green) in HCC tissues. MSI2 was co-localized with Notch1 in HCC tissues. Scale bar, 50 μm. **c** Immunofluorescence images of CD44v6+ SNU-398 cells and CD44v6- cells for localization of MSI2 (red) and Notch1 (green). Histogram analysis of the relative fluorescence intensity of MSI2 and Notch1 in CD44v6+ cells and CD44v6- cells. Scale bar, 50 μm. **d.** Silencing MSI2 decreased the expression of Notch1 receptor and Notch1 pathway target genes in CD44v6+ SNU-398 cells. β-actin was used as a normalized control. **e** Western blot showed that overexpression of MSI2 increased the expression of Notch1 receptor and Notch1 pathway target genes in CD44v6- SNU-398 cells. β-actin was used as a normalized control

Notch signaling was reported to be involved in MSI2 maintenance of the stemness in hematological malignancies [29]. To further investigate the correlation of MSI2 and Notch1 signaling in HCC, we explored the external dataset from the starBase v3.0 project which contains 374 HCC samples and 50 normal samples. The regression analysis showed that MSI2 was positively related to Notch1 in clinical-pathology (Fig. 5a, *r* = 0.458, *p*-value =8.02e-21). Moreover, the co-localization of MSI2 (red) and Notch1 (green) was observed by immunofluorescence staining in HCC tumors (Fig. 5b). These results suggested that MSI2 was positively correlated with Notch1 signaling pathway in HCC pathology.

Consistent with these results, immunofluorescence showed more Notch1 (green) and MSI2 (red) co-localization in CD44v6+ cells than CD44v6- cells. MSI2 (red) and Notch1 (green) co-localized in the cytoplasm and nucleus (Fig. 5c). MSI2 shRNA1 was more efficient than MSI2 shRNA2 and was used to suppress MSI2 expression in CD44v6+ cells. We found that repression of MSI2 in CD44v6+ cells decreased the expression of Notch1 receptor and Notch1 target genes also decreased (Fig. 5d). In accordance with this, overexpression of MSI2 in CD44v6- HCC cells increased the expression of Notch1 receptor and target genes (Fig. 5e). Collectively, these results indicated that Notch1 signaling pathway was downstream of MSI2 and MSI2 may maintain the stemness properties of CD44v6+ LCSCs through Notch1 signaling pathway.

### MSI2 required LFNG to activate Notch1 signaling pathway in CD44v6+ LCSCs

**Fig. 6:**
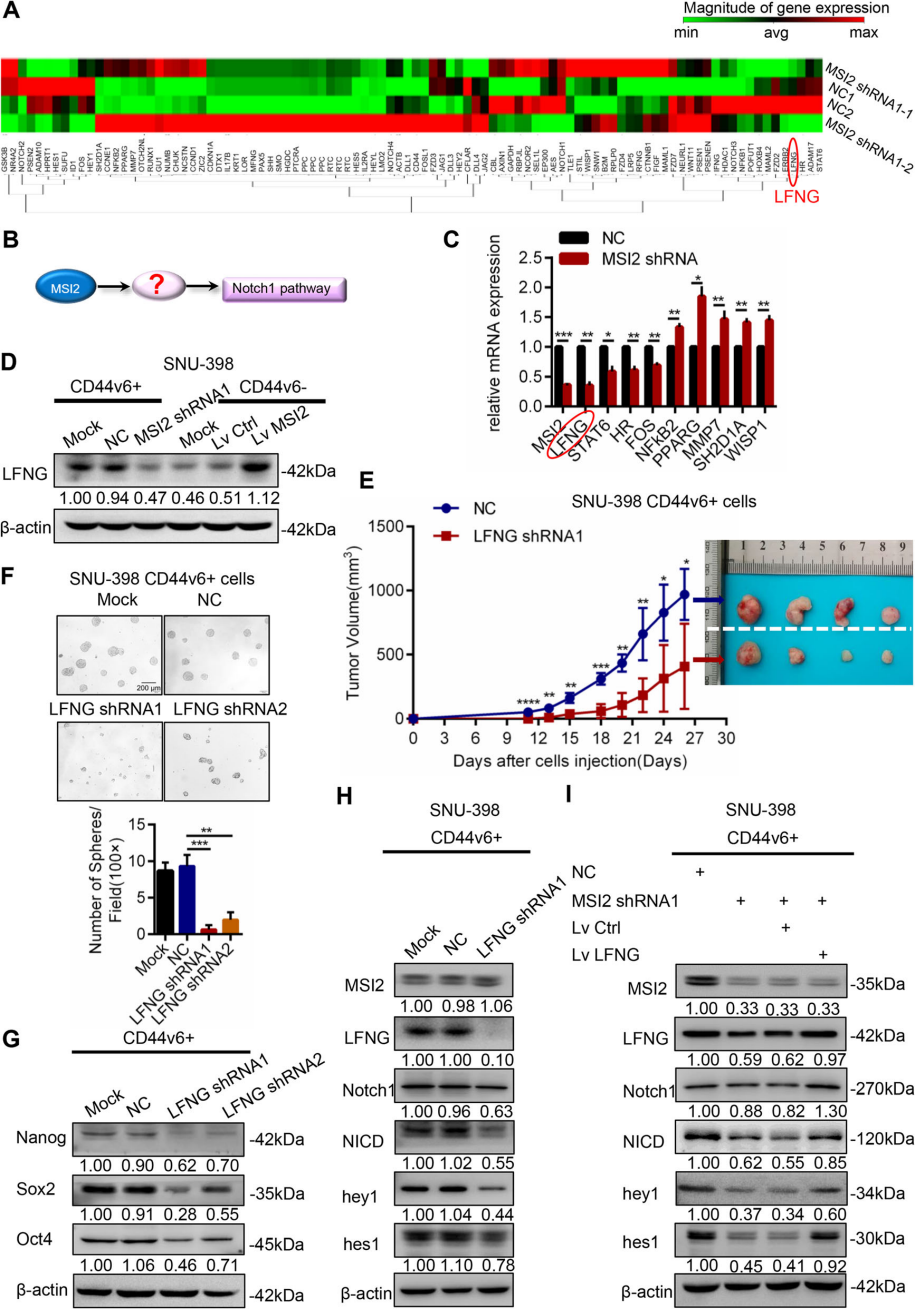
MSI2 activated Notch1 signaling through LFNG in CD44v6+ LCSCs. **a** A Notch RT^2^ PCR Array was used to determine mRNA expression profiles between MSI2 shRNA and control CD44v6+ LCSCs. **b** Hypothesis diagram of MSI2 regulates Notch1 signaling pathway. **c** Relative mRNA of the most significantly regulated genes were detected by RT-PCR in MSI2 shRNA1 group and the corresponding control group. **d** Western blot showed that silencing MSI2 decreased the expression of LFNG in CD44v6+ cells while overexpression of MSI2 increased the expression of LFNG in CD44v6- cells. β-actin was used as a normalized control. **e** Efficiency of tumor formation of LFNG shRNA1 cells and the corresponding controls. Number of injected cells: 1 × 10^5^. *n* = 4. **f** Representative images of spheres and histogram analysis in indicated cells. The inhibition of LFNG decreased self-renewal property in vitro in CD44v6+ LCSCs, Scale bar, 200 μm. **g** The expression of cancer stemness-related genes, including Nanog, Oct4 and Sox2 in LFNG shRNA cells compared with corresponding controls. β-actin was used as a normalized control. The inhibition of LFNG decreased the expression of stemness-related genes in CD44v6+ LCSCs. **h** Silencing LFNG in CD44v6+ HCC cells decreased the expression of key components of Notch1 pathway (including Notch1, NICD, Hey1 and Hes1) but MSI2 had no significant change. β-actin was used as a normalized control. **i** Key components of Notch1 signaling reduction caused by MSI2 knockdown could be rescued by LFNG overexpression in CD44v6+ LCSCs. For statistical analysis, *p < 0.05, **p < 0.01, ***p < 0.001 and ****p < 0.0001, t test

To further investigate the molecular mechanism of MSI2 in Notch1 activation, we compared the mRNA expression profiles between MSI2 shRNA1 and control treated CD44v6+ LCSCs using a Notch RT^2^ PCR Array, which contains 84 Notch pathway-focused genes as well as five housekeeping genes (Fig. 6a, QIAGEN, PAHS-059Z RT^2^ PCR Array). A total of 18 genes showed significantly differential expression (fold change ≥2, *P* ≤ 0.05) between the MSI2 shRNA1 group and the control group. Of these, 12 were upregulated and 6 were downregulated in the MSI2 shRNA1 expression group compared to the control group (Additional file 3: Figure S5B, Additional file 4: Table S2). Among these 18 target genes regulated by MSI2, we focus on the molecular which may regulate Notch1 signaling (Fig. 6b). We choose the most significantly differential expressed 10 genes (LFNG, STAT6, HR, FOS, NFKB2, PPARG, MMP7, SH2D1A, WISP1, HPRT1 (HPRT1 is housekeeping gene)) to validate by RT-PCR. The results showed that LFNG was downregulated by over 2-fold in the MSI2 shRNA1 group compared to the control group and was the most significantly regulated gene (Fig. 6c). LFNG was known to be a Notch1 receptor glycosyltransferase that regulates the expression of Notch1 receptor. LFNG catalyzes the addition of N-acetyl-glucosamine onto O-fucose residue on epidermal growth factor (EGF) repeats of the Notch1 receptor to alter signaling. This finding was also verified by western blot which found that LFNG was downregulated when the MSI2 was knocked down in CD44v6+ LCSCs whereas LFNG was upregulated when the MSI2 was overexpressed in CD44v6- cells (Fig. 6d).

Next, to confirm the contribution of LFNG in stemness properties including self-renewal and tumorigenic capacity of CD44v6+ LCSCs, we performed subcutaneous xenografts in NOD/SCID mice and spheroid formation assays. The results showed that the tumorigenicity of CD44v6+ HCC cells was inhibited when LFNG was downregulated with LFNG shRNA1 (Fig. 6e, 1× 10^5^ cells). Down-regulation of LFNG using two different LFNG shRNA also reduced the number of spheroids in CD44v6+ HCC cells (Fig. 6f). Moreover, the inhibition of LFNG by using LFNG shRNA1 and LFNG shRNA2 decreased the expression of stemness-related genes in CD44v6+ LCSCs (Fig. 6g).

Then, we explored the MSI2/LFNG/Notch1 axis in CD44v6+ LCSCs. Western blot showed that Notch1 signaling was inhibited in LFNG shRNA1 cells but MSI2 had no significant changes (Fig. 6h). However, overexpression of LFNG in CD44v6- HCC cells increased the expression of Notch1 receptor, Notch1 target genes, while MSI2 also had no significant change (Additional file 3: Figure S5C). These results indicated that LFNG was downstream of MSI2 but upstream of the Notch1 pathway. Additionally, expressions of the Notch1 receptor and Notch1 targets reduction caused by MSI2 knockdown were reversed by LFNG overexpression in CD44v6+ LCSCs (Fig. 6i). Moreover, the activation of Notch1 signaling caused by overexpression of MSI2 was reduced by LFNG downregulation in CD44v6- cells (Additional file 3: Figure S5D). Collectively, these data indicated that MSI2 activated Notch1 signaling through LFNG in CD44v6+ LCSCs.

### MSI2 bound to LFNG mRNA and protein directly

**Fig. 7:**
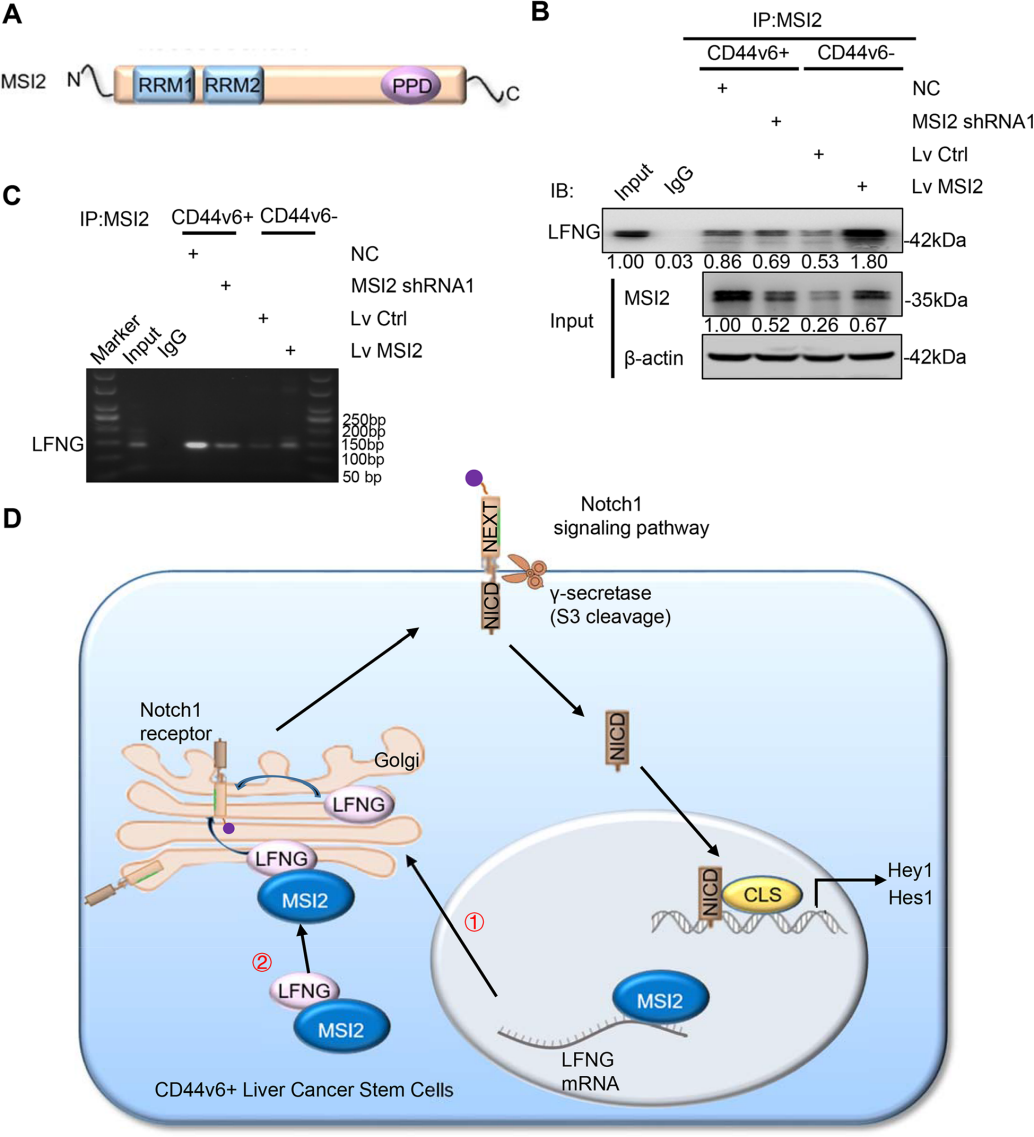
MSI2 bound to LFNG mRNA and protein directly. **a** Schematic representation of MSI2 molecular interaction domains for interaction with RNA and with proteins. RRM, RNA recognition motif; PPD, protein-protein binding domain. **b** CD44v6+ cells and CD44v6- cells were isolated from SNU-398 cells. CD44v6+ cells were transfected with MSI2 shRNA1 or corresponding control virus, CD44v6- cells were transfected with Lv MSI2 or corresponding control virus. Lysates were precipitated with anti-MSI2 antibody and then immunoblotted (WB) for LFNG. Protein expression of MSI2 and LFNG was also analyzed. **c** CD44v6+ cells were transfected with MSI2 shRNA1 or corresponding control virus, CD44v6- cells were transfected with Lv MSI2 or corresponding control virus. RIP assays using anti-MSI2 antibody showed that MSI2 interacted with LFNG. The results of agarose electrophoresis of the PCR products were shown. **d** Schematic illustration the mechanism by which MSI2 activating Notch1 signaling pathway by binding to LFNG mRNA and protein directly

MSI2 protein contains two RNA recognition motifs (RRMs) which are separated by a short linker region [29] and an auxiliary domain that often mediates protein-protein interaction [30] (Fig. 7a). To further explore the mechanism of how MSI2 regulates LFNG in activating Notch1 signaling, co-immunoprecipitation (Co-IP) and RNA-immunoprecipitation (RIP) were performed in SNU-398 cells. First, Co-IP experiments indicated that MSI2 interacted with LFNG, suggesting a close relationship between these two proteins. Moreover, the binding of MSI2 and LFNG was more prominent in CD44v6+ cells than CD44v6- cells. Furthermore, MSI2 knockdown decreased the binding of MSI2 to LFNG in CD44v6+ cells while MSI2 overexpression increased the binding of MSI2 to LFNG in CD44v6- cells (Fig. 7b). MSI2 has been shown to directly bind to *ESR1* mRNA to maintain its stability and regulate gene function [31]. Since we found that MSI2 influenced the LFNG mRNA expression, we hypothesized that MSI2 may directly bind to LFNG mRNA to regulate its expression. We verified the interaction between MSI2 and LFNG mRNA by RIP with anti-MSI2 antibody. The results revealed that MSI2 could bind to LFNG mRNA and this binding was more obvious in CD44v6+ cells than CD44v6- cells. Furthermore, the binding between MSI2 and LFNG mRNA decreased when MSI2 was inhibited in CD44v6+ cells, but increased when MSI2 was overexpressed in CD44v6- cells (Fig. 7c). Taken together, these data demonstrated that MSI2 might directly bind to LFNG mRNA and protein to regulate LFNG expression.

## Discussion

Accumulating evidence indicates that tumor growth, metastasis, and chemoradiotherapy resistance are highly related to the presence of CSCs, identified by stem cell markers. CD44v6 is reported to be one of the generally acknowledged CSCs’ markers in colon and prostatic cancer [6, 7, 32]. In this study, we demonstrated that CD44v6+ HCC cells possessed stemness properties of self-renewal, migration, invasion, resistance to Sorafenib, and tumorigenic capacity and could be a marker of LCSCs. In HCC, CD44v6 is highly expressed in HCC tumors and is correlated with worse prognosis [33, 34]. Consistent with this, we found increased expression of CD44v6 predicted worse prognosis in HCC patients and high expression of CD44v6 correlated with advanced stage in this study. Taken together, we showed that CD44v6 could be a LCSCs marker and could be a prognostic factor for HCC patients.

CD44v6 has been reported to be involved in cell migration and metastasis by binding to hepatocyte growth factor (HGF), osteopontin (OPN), and activating MET signaling [6, 35]. Nevertheless, the molecular mechanism involved in maintaining the stemness properties of CD44v6+ LCSCs remains to be elucidated. In this study, we found MSI2 was positively related to CD44v6 in HCC and was elevated in CD44v6+ cells compared to CD44v6- cells, which indicated that MSI2 might act as a key regulator in the maintenance of CD44v6+ LCSCs. MSI2 has a pivotal role in stem cell function and cell fate determination. Firstly, MSI2 is identified in stem and progenitor cells that regulates differentiation [30, 36] and to be a critical regulator of hematopoietic stem cell self-renewal in myeloid leukemia and myelodysplastic syndrome [37, 38]. Recently, Fang et al. showed that MSI2 promoted self-renewal and drug resistance via LIN28A in HCC [28]. Consistent with these previous studies, we found that MSI2 was significantly overexpressed in human HCC tumors, and patients with higher expression of MSI2 had a comparatively poorer prognosis [18, 28]. Of note, we found MSI2 was up-regulated in CD44v6+ HCC cells compare to CD44v6- HCC cells and that MSI2 was co-localized with CD44v6. Here, we provided strong evidence that MSI2 could maintain the stemness properties of CD44v6+ LCSCs. Silencing MSI2 decreased CD44v6+ cells self-renewal, proliferation, migration, and invasion in vitro and tumorigenicity in vivo. On the contrary, CD44v6^−^-non-LCSCs regained stemness properties after overexpression MSI2. Taken together, these data suggested MSI2 is a key regulator involved in the maintenance of self-renewal and tumorigenesis of CD44v6+ LCSCs, and might open up new perspectives for treatment.

Increasing evidence indicates that Notch signaling can promote self-renewal and stem cell properties in HCC, glioblastoma, and other tumors [21, 39, 40]. Our previous studies also demonstrated that the Nocth1 pathway was important in the promotion and maintenance of LCSCs [25–27]. In our study, we extend this previous work to show that Notch1 signaling was activated in CD44v6+ LCSCs. Inhibition of Notch1 signaling in CD44v6+ cells significantly attenuated the stemness properties, decreased the expression of the stemness-related genes. Consequently, we further clarified the key role of Notch1 signaling pathway in LCSCs, including CD44v6+ LCSCs.

The study of Ito and Griner, et al. showed that the Notch pathway inhibitor Numb is the main target of MSI2 in regulating Notch signaling pathway in hematologic malignancies [11, 41]. However, in solid tumors of HCC, our results did not show a significant difference in the expression of Numb when MSI2 was downregulated in CD44v6+ LCSCs or upregulated in CD44v6- HCC cells (Additional file 3: Figure S5A). This could be interpreted to show that Numb was not the main regulator to activate Notch1 signaling pathway in CD44v6+ LCSCs. The variance may be related to specific tumors and cellular contexts. According to our result using the Notch RT^2^ PCR Array, we focus on LFNG, which is also known as O-fucosylpeptide 3-beta-N-acetylglucosaminyltransferase, a regulator of Notch signaling. LFNG catalyzes the addition of N-acetyl-glucosamine onto *O*-fucose residue on epidermal growth factor (EGF) repeats of the Notch1 receptor to alter signaling [24]. The effect of LFNG in regulating Notch signaling pathway is controversial. For instance, in *KRAS*-induced pancreatic ductal adenocarcinoma (PDAC) the deletion of LFNG caused the activation of Notch signaling and accelerated PDAC development [42]. Similar studies have also been reported in claudin-low breast cancer (CLBC) and prostate cancer [43, 44]. These studies demonstrated LFNG is negatively related to Notch signaling. Other studies showing that LFNG is positively related to Notch signaling during T cell differentiation and brain development [45, 46]. The opposite effects may be related to the different Notch ligands, LFNG enhances Notch activation by Delta-like (DLL) family ligands and diminishes Notch activation by Jagged family ligands [24]. In our study, we found LFNG could be regulated by MSI2 and was positively related to Notch1 signaling. Moreover, we verified its role for maintaining self-renewal and tumorigenicity of CD44v6+ LCSCs. Collectively, our finding confirmed the role of LFNG in the maintenance of CD44v6+ LCSCs and revealed MSI2 activate Notch1 signaling through LFNG.

MSI2 protein has two RNA recognition motifs (RRMs) separated by a short linker region and an auxiliary domain that often mediates protein-protein interaction, which mean MSI2 could bind to RNA as well as protein [29]. MSI2 has been reported to maintain protein and RNA stability to regulate ESR1 function in breast cancer [31]. Fang et al. also showed that MSI2 could directly bind to LIN28A to regulate the stemness property and chemoresistance of LCSCs [28]. Here, we found MSI2 could bind to LFNG protein and the binding showed more in CD44v6+ LCSCs than CD44v6- cells. Furthermore, we found MSI2 could also directly bind to LFNG mRNA to regulate its expression. This evidence suggested that MSI2 regulated LFNG expression and function by both binding to the LFNG protein and the LFNG mRNA. Our finding revealed a new molecular mechanism of Notch1 signaling activated by MSI2.

## Conclusions

Our study demonstrated that MSI2 was positively related to CD44v6 and poor prognosis. Additionally, MSI2 was highly expressed in CD44v6+ LCSCs and maintained the stemness properties of CD44v6+ LCSCs. Mechanically, MSI2 bound to LFNG mRNA and protein directly to regulate the expression of Notch1 receptor in Golgi, then activated Notch1 signaling pathway, contributing the maintenance of stemness of CD44v6+ LCSCs (shown in Fig. 7d). Our findings provide a new insight to the recurrence and metastasis of HCC and potential molecular targets for targeted therapy of liver cancer.

## Supplementary information

**Additional file 1: Figure S1** A. Western blot analysis of CD44v6 and MSI2 protein levels in HCC tissues and adjacent non-tumor tissues selected randomly. β-actin was used as a normalized control. B, C and D. IHC staining for CD44v6 and MSI2 and intensity of staining was evaluated as described in methods. For each marker, representative images were shown demonstrating each of intensity grades of staining. Scale bar, 50 μm. **Figure S2** A and B. CD44v6 expression levels varied in human hepatic L02 cells and HCC cell lines (HepG2, MHCC-97L, SMMC-7721, HLE, Huh-7, MHCC-97h and SNU-398) by flow cytometry analysis. C. Representative FACS of CD44v6+ and CD44v6- populations, which isolated from SNU-398 and MHCC-97h cell lines by magnetic bead sorting.

**Additional file 2: Figure S3** A. Overexpression of MSI2 increased self-renewal property in vitro in CD44v6- SNU-398 cells, Scale bar, 200 μm. B and C. Transwell migration and invasion assays showed that up-regulation of MSI2 increased the migration and invasion capacity of CD44v6- SNU-398 cells. Scale bar, 200 μm. D. Colony formation assays showed the ability of cell proliferation and colony formation of CD44v6- SNU-398 cells was enhanced when MSI2 was up-regulated. E. 1×10^5^ of Lv MSI2 cells and the corresponding controls were injected into the left lobes of liver. Bioluminescence signals from Lv MSI2 group were stronger than those from the corresponding control group. n=6. F. Overexpression of MSI2 increased the expression of stemness-related genes in CD44v6- SNU-398 cells. G. CCK8 toxic assay showed that MSI2 shRNA cells were less resistant to Sorafenib than the control cells. H. RT-PCR showed that the inhibition of MSI2 decreased the expression of stemness-related genes in CD44v6+ SNU-398 cells. For statistical analysis, *p < 0.05, **p < 0.01, ***p < 0.001 and ****p < 0.0001, t test. **Figure S4** A. Notch1 signaling pathway was inhibited by Notch1 shRNA lentivirus. B. Notch1 signaling pathway was inhibited by γ-secretase inhibitor RO4929097. C. The inhibition of Notch1 signaling decreased self-renewal property in vitro in CD44v6+ SNU-398 cells, Scale bar, 200 μm. D and E. Transwell migration and invasion assay showed that the inhibition of Notch1 signaling decreased the migration and invasion capacity of CD44v6+ SNU-398 cells. Scale bar, 200 μm. F. Colony formation assays showed that the ability of cell proliferation and colony formation of CD44v6+ SNU-398 cells was inhibited when Notch1 signaling was inhibited. G. The inhibition of Notch1 signaling in CD44v6+ SNU-398 cells decreased the expression of stemness-related genes. For statistical analysis, **p < 0.01 and ***p < 0.001, t test.

**Additional file 3: Figure S5** A. Western blot showed that the expression of Numb had no significant difference when MSI2 was down-regulated in CD44v6+ cells or up-regulated in CD44v6- cells. B. Significantly differential expression genes (fold change ≥2, p≤0.05) between MSI2 shRNA 1 group and control group. Blue histogram represented down-regulated genes and the red represented up-regulated genes in the MSI2 shRNA 1 group compared to the control group. C. Western blot showed that overexpression of LFNG in CD44v6- HCC cells increased the expression of key components of Notch1 pathway (including Notch1, NICD, Hey1 and Hes1) but MSI2 had no significant change. β-actin was used as a normalized control. D. Western blot showed that the activation of Notch1 signaling caused by MSI2 overexpression could be inhibited by LFNG silencing in CD44v6- cells. E. LFNG protein levels in LFNG shRNA cells compared with corresponding control cells. β-actin was used as a normalized control. **Figure S6** The result of positive control (SNRNP70) and negative control (U1) of RIP assays.

**Additional file 4: Table S1.** Tumor engraftment rates of HCC cells. **Table S2.** Significantly differential genes (fold change ≥2, *p* ≤ 0.05) between MSI2 shRNA groups and control groups in CD44v6+ cells **Table S3.** Primer sequences used to amplify specific target genes

## Data Availability

All data generated or analyzed during this study are included in this published article and its supplementary information files.

## Abbreviations

CCK-8: Cell counting kit-8
Co-IP: Co-immunoprecipitation
FBS: Fetal bovine serum
HCC: Hepatocellular carcinoma
IB: Immunoblotting
IHC: Immunohistochemistry
LCSCs: Liver cancer stem cells
LFNG: LFNG O-fucosylpeptide 3-beta-N-acetylglucosaminyltransferase
MACS: Magnetic activated cell sorting
NOD-SCID: Non-obese, diabetic, severe combined immunodeficiency
PCR: Polymerase chain reaction
PPD: Protein-protein binding domain
RRM: RNA recognition motif
shRNA: Short hairpin RNA
