# Supplementary material for: Musashi2 contributes to the maintenance of CD44v6+ liver cancer stem cells via notch1 signaling pathway
Source: J Exp Clin Cancer Res. 2019 Dec 30;38:505. doi: 10.1186/s13046-019-1508-1 (PMC6936093; doi:10.1186/s13046-019-1508-1)
Supplement: Supplementary file 1 — Additional file 1: Figure S1 A. Western blot analysis of CD44v6 and MSI2 protein levels in HCC tissues and adjacent non-tumor tissues selected randomly. β-actin was used as a normalized control. B, C and D. IHC staining for CD44v6 and MSI2 and intensity of staining was evaluated as described in methods. For each marker, representative images were shown demonstrating each of intensity grades of staining. Scale bar, 50 μm. Figure S2 A and B. CD44v6 expression levels varied in human hepatic L02 cells and HCC cell lines (HepG2, MHCC-97L, SMMC-7721, HLE, Huh-7, MHCC-97h and SNU-398) by flow cytometry analysis. C. Representative FACS of CD44v6+ and CD44v6- populations, which isolated from SNU-398 and MHCC-97h cell lines by magnetic bead sorting. [file 13046_2019_1508_MOESM1_ESM.docx]

**Supplementary Material:**

**Musashi2 Contributes to the Maintenance of CD44v6+ Liver Cancer Stem Cells via Notch1 Signaling Pathway**

Xiju Wang ^1,†^, Ronghua Wang ^1,†^, Shuya Bai ^1^, Si Xiong ^1^, Yawen Li ^1^, Man Liu ^1^, Zhenxiong Zhao ^1^, Yun Wang ^1^, Yuchong Zhao ^1^, Wei Chen ^1^, Timothy R. Billiar ^2^, Bin Cheng ^1 *^

^1^ Department of Gastroenterology and Hepatology, Tongji Hospital, Tongji Medical College, Huazhong University of Science and Technology, Wuhan, PR China 430030.

^2^ Department of Surgery, University of Pittsburgh School of Medicine, Pittsburgh, PA 15213

^†^ Xiju Wang, Ronghua Wang contributed equally to this work.

***Corresponding Author:** Bin Cheng, Department of Gastroenterology and Hepatology, Tongji Hospital, Tongji Medical College, Huazhong University of Science and Technology, Wuhan, PR China. Tel: +86-27 69378505; Fax: +86-27 69378505; E-mail address: [b.cheng@tjh.tjmu.edu.cn](mailto:b.cheng@tjh.tjmu.edu.cn).

**Contents**

**Supplementary Figure S1**

**Supplementary Figure S2**

**Figure S1**

**
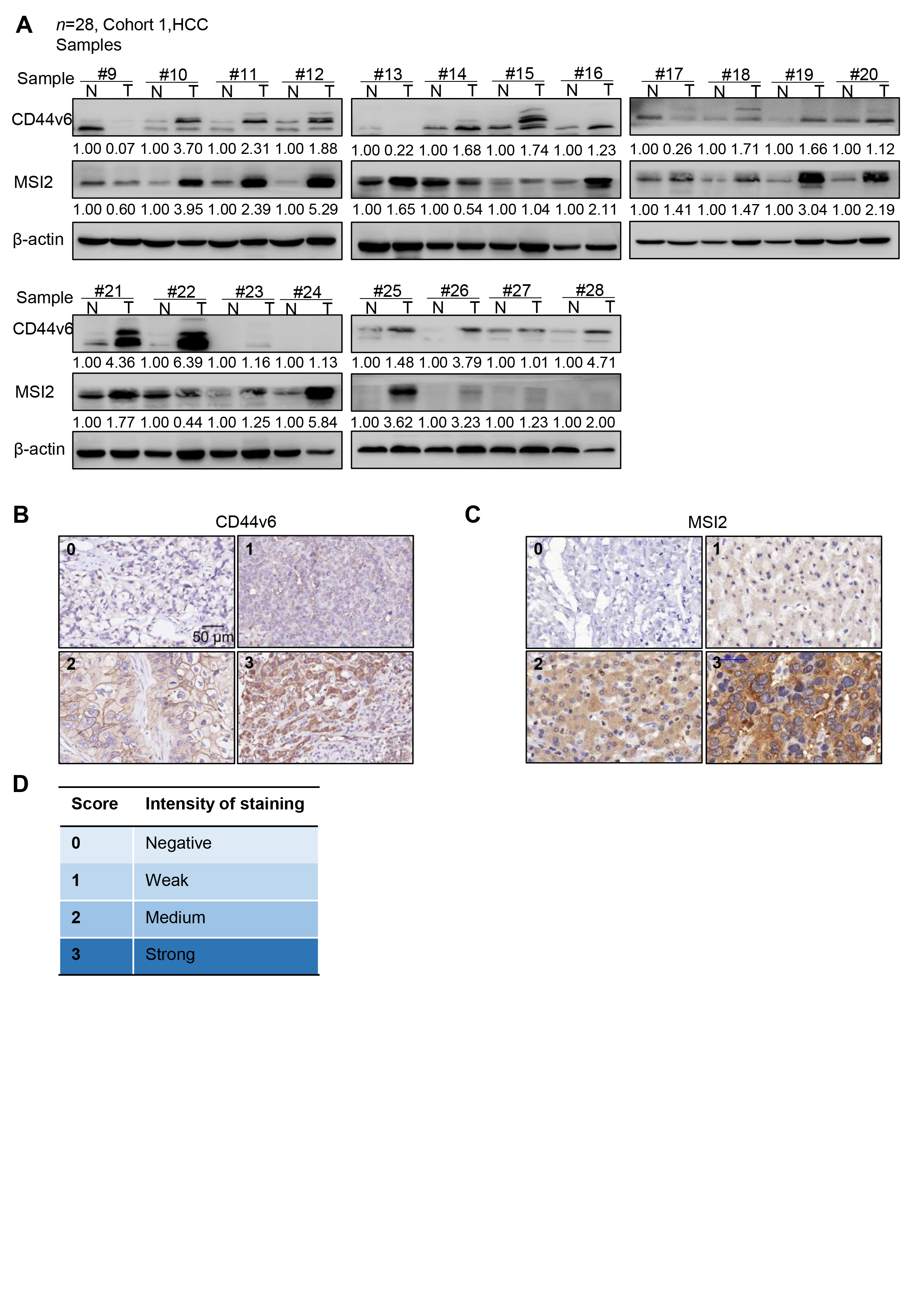
**

**Figure S1 A.** Western blot analysis of CD44v6 and MSI2 protein levels in HCC tissues and adjacent non-tumor tissues selected randomly. β-actin was used as a normalized control. **B, C and D.** IHC staining for CD44v6 and MSI2 and intensity of staining was evaluated as described in methods. For each marker, representative images were shown demonstrating each of intensity grades of staining. Scale bar, 50 μm.

**Figure S2**

**
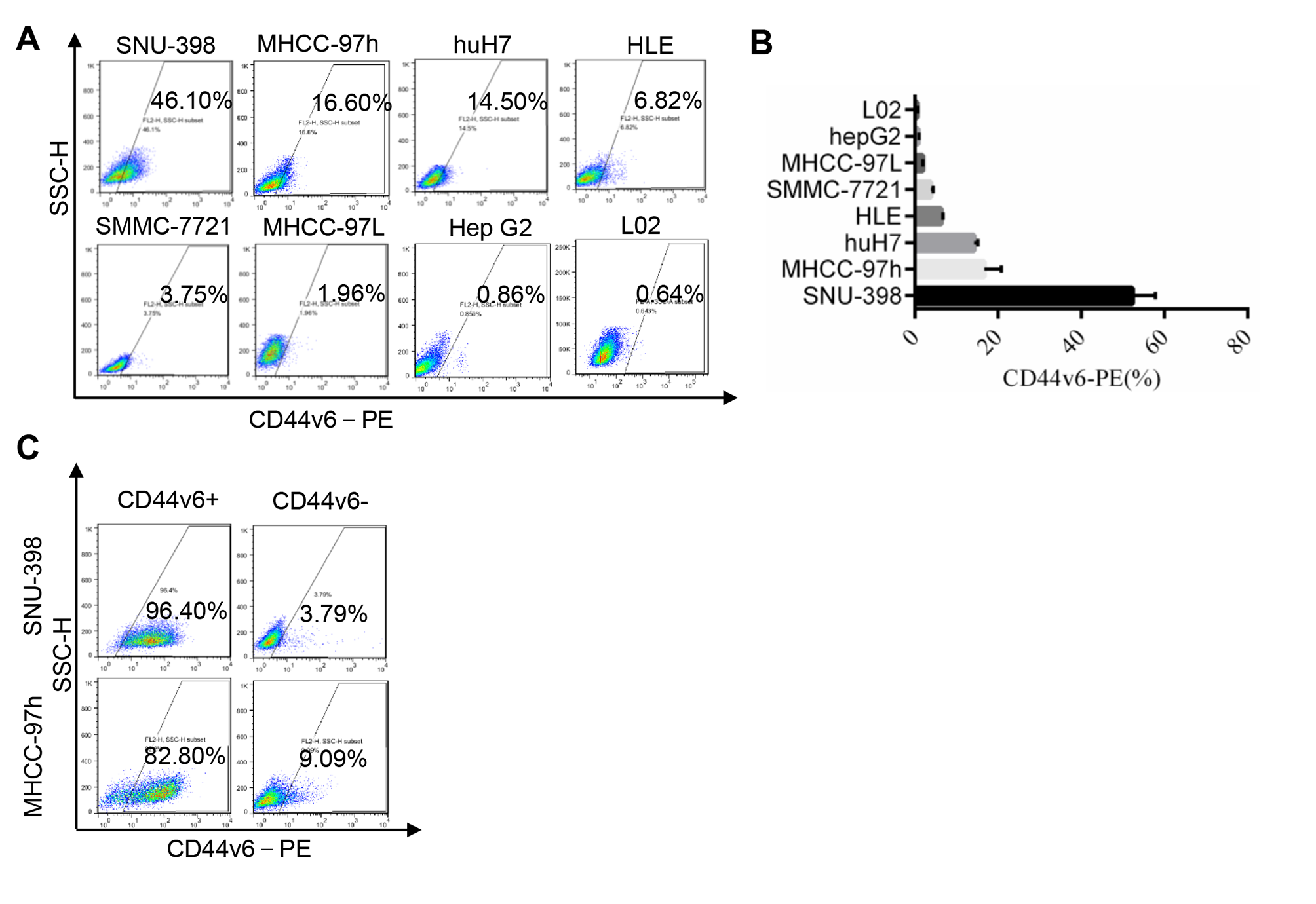
**

**Figure S2 A and B.** CD44v6 expression levels varied in human hepatic L02 cells and HCC cell lines (HepG2, MHCC-97L, SMMC-7721, HLE, Huh-7, MHCC-97h and SNU-398) by flow cytometry analysis. **C.** Representative FACS of CD44v6+ and CD44v6- populations, which isolated from SNU-398 and MHCC-97h cell lines by magnetic bead sorting.
