# Supplementary material for: Musashi2 contributes to the maintenance of CD44v6+ liver cancer stem cells via notch1 signaling pathway
Source: J Exp Clin Cancer Res. 2019 Dec 30;38:505. doi: 10.1186/s13046-019-1508-1 (PMC6936093; doi:10.1186/s13046-019-1508-1)
Supplement: Supplementary file 2 — Additional file 2: Figure S3 A. Overexpression of MSI2 increased self-renewal property in vitro in CD44v6- SNU-398 cells, Scale bar, 200 μm. B and C. Transwell migration and invasion assays showed that up-regulation of MSI2 increased the migration and invasion capacity of CD44v6- SNU-398 cells. Scale bar, 200 μm. D. Colony formation assays showed the ability of cell proliferation and colony formation of CD44v6- SNU-398 cells was enhanced when MSI2 was up-regulated. E. 1×10^5^ of Lv MSI2 cells and the corresponding controls were injected into the left lobes of liver. Bioluminescence signals from Lv MSI2 group were stronger than those from the corresponding control group. n=6. F. Overexpression of MSI2 increased the expression of stemness-related genes in CD44v6- SNU-398 cells. G. CCK8 toxic assay showed that MSI2 shRNA cells were less resistant to Sorafenib than the control cells. H. RT-PCR showed that the inhibition of MSI2 decreased the expression of stemness-related genes in CD44v6+ SNU-398 cells. For statistical analysis, *p < 0.05, **p < 0.01, ***p < 0.001 and ****p < 0.0001, t test. Figure S4 A. Notch1 signaling pathway was inhibited by Notch1 shRNA lentivirus. B. Notch1 signaling pathway was inhibited by γ-secretase inhibitor RO4929097. C. The inhibition of Notch1 signaling decreased self-renewal property in vitro in CD44v6+ SNU-398 cells, Scale bar, 200 μm. D and E. Transwell migration and invasion assay showed that the inhibition of Notch1 signaling decreased the migration and invasion capacity of CD44v6+ SNU-398 cells. Scale bar, 200 μm. F. Colony formation assays showed that the ability of cell proliferation and colony formation of CD44v6+ SNU-398 cells was inhibited when Notch1 signaling was inhibited. G. The inhibition of Notch1 signaling in CD44v6+ SNU-398 cells decreased the expression of stemness-related genes. For statistical analysis, **p < 0.01 and ***p < 0.001, t test. [file 13046_2019_1508_MOESM2_ESM.docx]

**Supplementary Material:**

**Musashi2 Contributes to the Maintenance of CD44v6+ Liver Cancer Stem Cells via Notch1 Signaling Pathway**

Xiju Wang ^1,†^, Ronghua Wang ^1,†^, Shuya Bai ^1^, Si Xiong ^1^, Yawen Li ^1^, Man Liu ^1^, Zhenxiong Zhao ^1^, Yun Wang ^1^, Yuchong Zhao ^1^, Wei Chen ^1^, Timothy R. Billiar ^2^, Bin Cheng ^1 *^

^1^ Department of Gastroenterology and Hepatology, Tongji Hospital, Tongji Medical College, Huazhong University of Science and Technology, Wuhan, PR China 430030.

^2^ Department of Surgery, University of Pittsburgh School of Medicine, Pittsburgh, PA 15213

^†^ Xiju Wang, Ronghua Wang contributed equally to this work.

***Corresponding Author:** Bin Cheng, Department of Gastroenterology and Hepatology, Tongji Hospital, Tongji Medical College, Huazhong University of Science and Technology, Wuhan, PR China. Tel: +86-27 69378505; Fax: +86-27 69378505; E-mail address: [b.cheng@tjh.tjmu.edu.cn](mailto:b.cheng@tjh.tjmu.edu.cn).

**Contents**

**Supplementary Figure S3**

**Supplementary Figure S4**

**Figure S3**


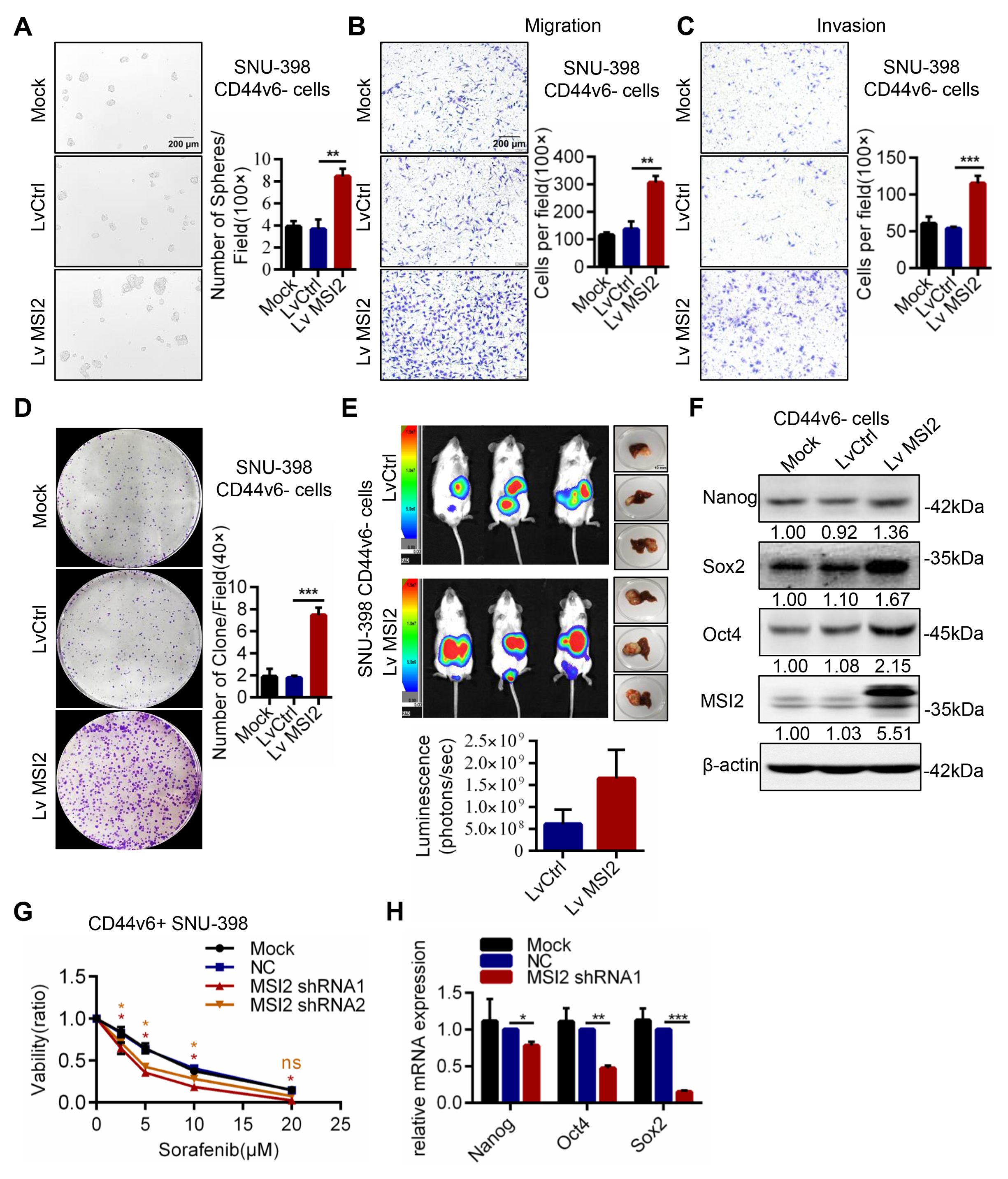


**Figure S3 A.** Representative images of spheres and histogram analysis in indicated cells. Overexpression of MSI2 increased self-renewal property *in vitro* in CD44v6- SNU-398 cells, Scale bar, 200 μm. **B and C.** Transwell migration and invasion assays showed that up-regulation of MSI2 increased the migration and invasion capacity of CD44v6- SNU-398 cells. Scale bar, 200 μm. **D.** Colony formation assays showed the ability of cell proliferation and colony formation of CD44v6- SNU-398 cells was enhanced when MSI2 was up-regulated. **E.** 1×10^5^ of Lv MSI2 cells and the corresponding controls were injected into the left lobes of liver. Bioluminescence signals from Lv MSI2 group were stronger than those from the corresponding control group. *n*=6. **F.** The expression of cancer stemness-related genes, including Nanog, Oct4 and Sox2 in Lv MSI2 cells compared with corresponding control. β-actin was used as a normalized control. Overexpression of MSI2 increased the expression of stemness-related genes in CD44v6- SNU-398 cells. **G.** MSI2 shRNA cells and control cells were treated with Sorafenib for 24 h and evaluated by CCK8 toxic assay. It showed that MSI2 shRNA cells were less resistant to Sorafenib than the control cells. **H.** RT-PCR showed that the inhibition of MSI2 decreased the expression of stemness-related genes in CD44v6+ SNU-398 cells. For statistical analysis, **p* < 0.05, ***p* < 0.01, ****p* < 0.001 and *****p* < 0.0001, t test.

**Figure S4**


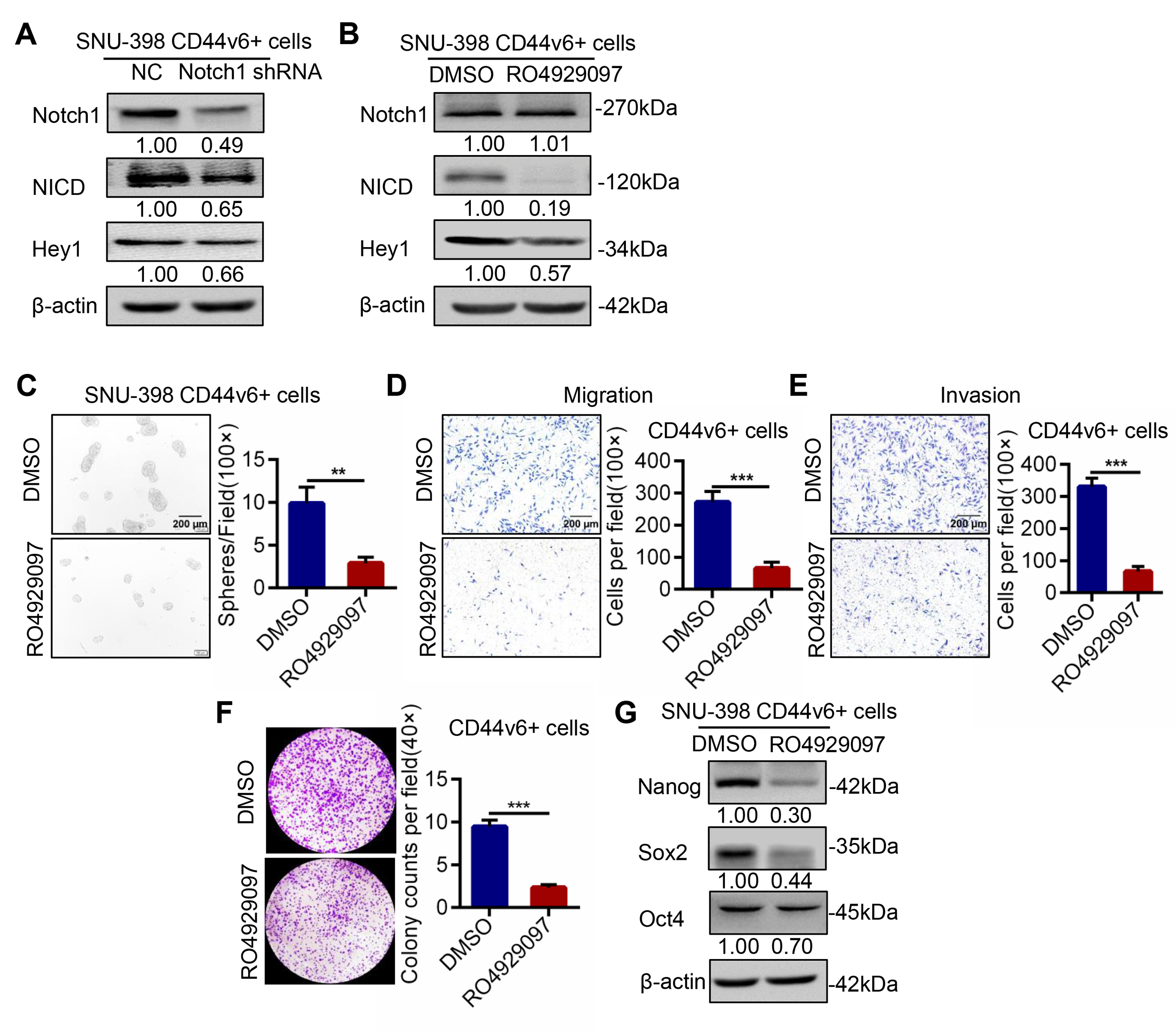


**Figure S4 A.** Notch1 signaling pathway was inhibited by Notch1 shRNA lentivirus. **B.** Notch1 signaling pathway was inhibited by γ-secretase inhibitor RO4929097. **C.** Representative images of spheres and histogram analysis in indicated cells. The inhibition of Notch1 signaling decreased self-renewal property *in vitro* in CD44v6+ SNU-398 cells, Scale bar, 200 μm. **D and E.** Transwell migration and invasion assay showed that the inhibition of Notch1 signaling decreased the migration and invasion capacity of CD44v6+ SNU-398 cells. Scale bar, 200 μm. **F.** Colony formation assays showed that the ability of cell proliferation and colony formation of CD44v6+ SNU-398 cells was inhibited when Notch1 signaling was inhibited. **G.** The inhibition of Notch1 signaling in CD44v6+ SNU-398 cells decreased the expression of stemness-related genes. For statistical analysis, ***p* < 0.01 and ****p* < 0.001, t test.
