# Supplementary material for: Musashi2 contributes to the maintenance of CD44v6+ liver cancer stem cells via notch1 signaling pathway
Source: J Exp Clin Cancer Res. 2019 Dec 30;38:505. doi: 10.1186/s13046-019-1508-1 (PMC6936093; doi:10.1186/s13046-019-1508-1)
Supplement: Supplementary file 3 — Additional file 3: Figure S5 A. Western blot showed that the expression of Numb had no significant difference when MSI2 was down-regulated in CD44v6+ cells or up-regulated in CD44v6- cells. B. Significantly differential expression genes (fold change ≥2, p≤0.05) between MSI2 shRNA 1 group and control group. Blue histogram represented down-regulated genes and the red represented up-regulated genes in the MSI2 shRNA 1 group compared to the control group. C. Western blot showed that overexpression of LFNG in CD44v6- HCC cells increased the expression of key components of Notch1 pathway (including Notch1, NICD, Hey1 and Hes1) but MSI2 had no significant change. β-actin was used as a normalized control. D. Western blot showed that the activation of Notch1 signaling caused by MSI2 overexpression could be inhibited by LFNG silencing in CD44v6- cells. E. LFNG protein levels in LFNG shRNA cells compared with corresponding control cells. β-actin was used as a normalized control. Figure S6 The result of positive control (SNRNP70) and negative control (U1) of RIP assays. [file 13046_2019_1508_MOESM3_ESM.docx]

**Supplementary Material:**

**Musashi2 Contributes to the Maintenance of CD44v6+ Liver Cancer Stem Cells via Notch1 Signaling Pathway**

Xiju Wang ^1,†^, Ronghua Wang ^1,†^, Shuya Bai ^1^, Si Xiong ^1^, Yawen Li ^1^, Man Liu ^1^, Zhenxiong Zhao ^1^, Yun Wang ^1^, Yuchong Zhao ^1^, Wei Chen ^1^, Timothy R. Billiar ^2^, Bin Cheng ^1 *^

^1^ Department of Gastroenterology and Hepatology, Tongji Hospital, Tongji Medical College, Huazhong University of Science and Technology, Wuhan, PR China 430030.

^2^ Department of Surgery, University of Pittsburgh School of Medicine, Pittsburgh, PA 15213

^†^ Xiju Wang, Ronghua Wang contributed equally to this work.

***Corresponding Author:** Bin Cheng, Department of Gastroenterology and Hepatology, Tongji Hospital, Tongji Medical College, Huazhong University of Science and Technology, Wuhan, PR China. Tel: +86-27 69378505; Fax: +86-27 69378505; E-mail address: [b.cheng@tjh.tjmu.edu.cn](mailto:b.cheng@tjh.tjmu.edu.cn).

**Contents**

**Supplementary Figure S5**

**Supplementary Figure S6**

**Figure S5**


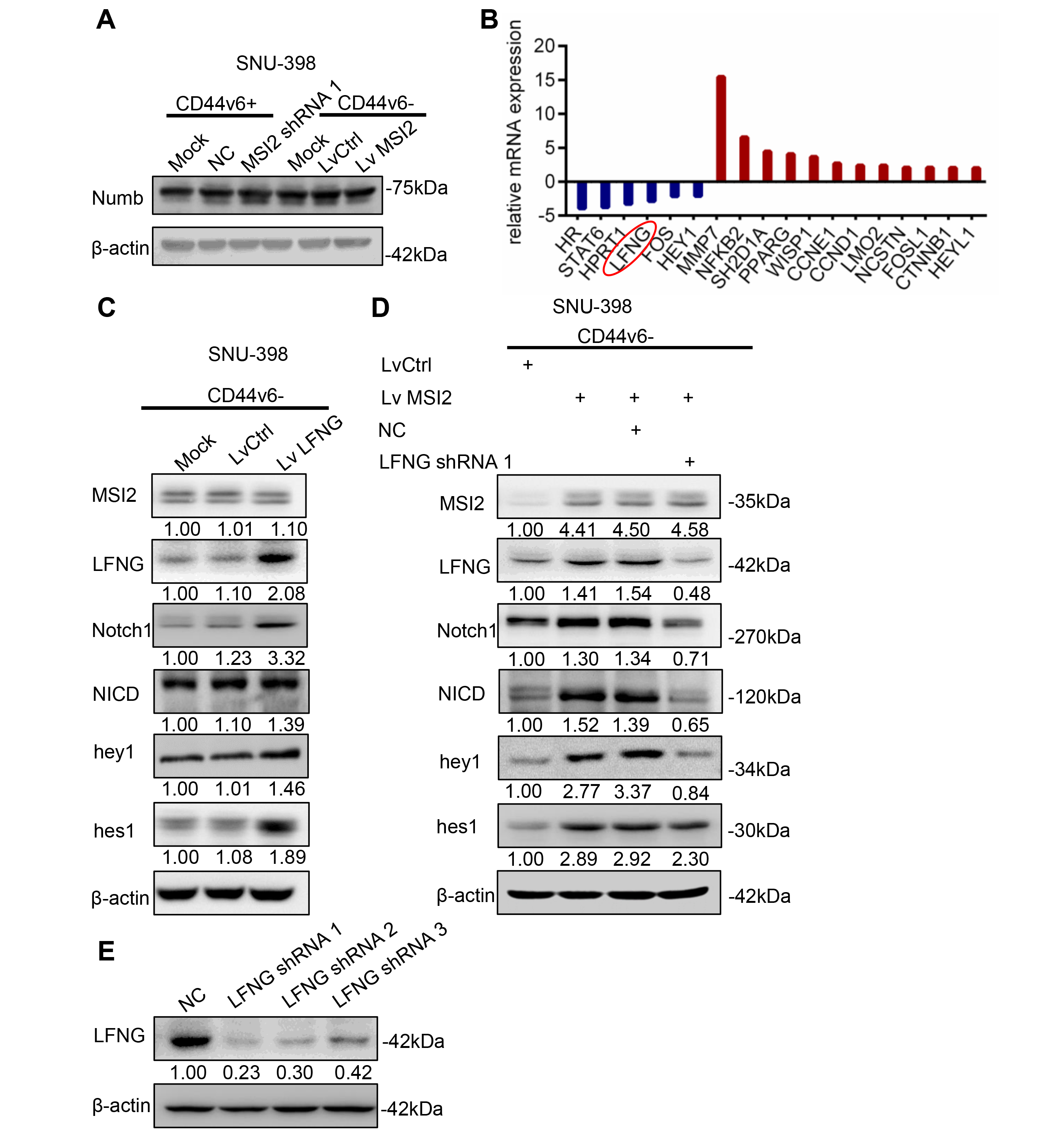


**Figure S5 A.** Western blot showed that the expression of Numb had no significant difference when MSI2 was down-regulated in CD44v6+ cells or up-regulated in CD44v6- cells. **B.** Significantly differential expression genes (fold change ≥2, p≤0.05) between MSI2 shRNA 1 group and control group. Blue histogram represented down-regulated genes and the red represented up-regulated genes in the MSI2 shRNA 1 group compared to the control group. **C.** Western blot showed that overexpression of LFNG in CD44v6- HCC cells increased the expression of key components of Notch1 pathway (including Notch1, NICD, Hey1 and Hes1) but MSI2 had no significant change. β-actin was used as a normalized control. **D.** Western blot showed that the activation of Notch1 signaling caused by MSI2 overexpression could be inhibited by LFNG silencing in CD44v6- cells. **E.** LFNG protein levels in LFNG shRNA cells compared with corresponding control cells. β-actin was used as a normalized control.

**Figure S6**


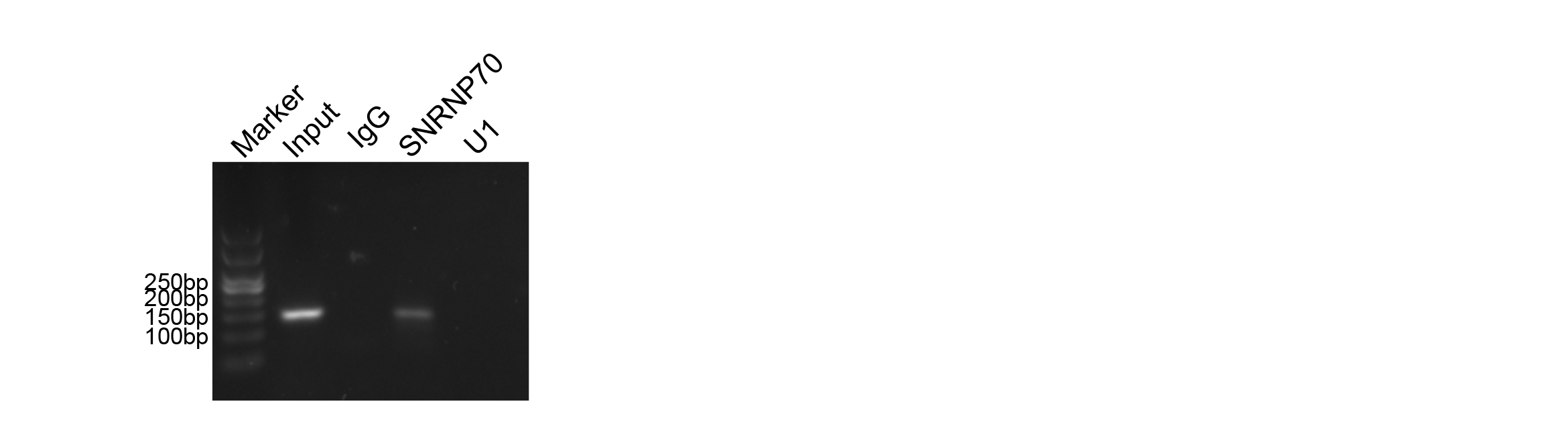


**Figure S6** The result of positive control (SNRNP70) and negative control (U1) of RIP assays.
