# Supplementary material for: Musashi2 contributes to the maintenance of CD44v6+ liver cancer stem cells via notch1 signaling pathway
Source: J Exp Clin Cancer Res. 2019 Dec 30;38:505. doi: 10.1186/s13046-019-1508-1 (PMC6936093; doi:10.1186/s13046-019-1508-1)
Supplement: Supplementary file 4 — Additional file 4: Table S1. Tumor engraftment rates of HCC cells. Table S2. Significantly differential genes (fold change ≥2, p ≤ 0.05) between MSI2 shRNA groups and control groups in CD44v6+ cells Table S3. Primer sequences used to amplify specific target genes [file 13046_2019_1508_MOESM4_ESM.docx]

**Supplementary Material:**

**Musashi2 Contributes to the Maintenance of CD44v6+ Liver Cancer Stem Cells via Notch1 Signaling Pathway**

Xiju Wang ^1,†^, Ronghua Wang ^1,†^, Shuya Bai ^1^, Si Xiong ^1^, Yawen Li ^1^, Man Liu ^1^, Zhenxiong Zhao ^1^, Yun Wang ^1^, Yuchong Zhao ^1^, Wei Chen ^1^, Timothy R. Billiar ^2^, Bin Cheng ^1 *^

^1^ Department of Gastroenterology and Hepatology, Tongji Hospital, Tongji Medical College, Huazhong University of Science and Technology, Wuhan, PR China 430030.

^2^ Department of Surgery, University of Pittsburgh School of Medicine, Pittsburgh, PA 15213

^†^ Xiju Wang, Ronghua Wang contributed equally to this work.

***Corresponding Author:** Bin Cheng, Department of Gastroenterology and Hepatology, Tongji Hospital, Tongji Medical College, Huazhong University of Science and Technology, Wuhan, PR China. Tel: +86-27 69378505; Fax: +86-27 69378505; E-mail address: [b.cheng@tjh.tjmu.edu.cn](mailto:b.cheng@tjh.tjmu.edu.cn).

**Contents**

**Supplementary Table S1**

**Supplementary Table S2**

**Supplementary Table S3**

**Table S1.** **Tumor engraftment rates of HCC cells**

| Cell numbers injected | Cell type | Tumor incidence^1^ | Latency (days)^2^ |
| --- | --- | --- | --- |
| 1×10^5^ | CD44v6+ SNU-398 | 4/4 | 7 |
|  | CD44v6- SNU-398 | 4/4 | 17 |
| 1×10^4^ | CD44v6+ SNU-398 | 4/4 | 13 |
|  | CD44v6- SNU-398 | 4/4 | 25 |
| 1×10^3^ | CD44v6+ SNU-398 | 4/4 | 19 |
|  | CD44v6- SNU-398 | 1/4 | 27 |

^1^. No. of Mice with Tumor Formation/Total No. of Mice with Cell Injection.

^2^. Approximate No. of days from tumor cell injection to the first appearance of tumors

**Table S2. Significantly differential genes (fold change ≥2, *p*≤0.05) between MSI2 shRNA groups and control groups in CD44v6+ cells**

| Gene Symbol | RefSeq | Fold Regulation | *p*-Value |
| --- | --- | --- | --- |
| Downregulated |  |  |  |
| LFNG | NM_001040167 | -2.86 | 0.000000 |
| FOS | NM_005252 | -2.12 | 0.000000 |
| HEY1 | NM_012258 | -2.11 | 0.000000 |
| HR | NM_018411 | -3.92 | 0.000000 |
| STAT6 | NM_003153 | -3.75 | 0.000000 |
| HPRT1 | NM_000194 | -3,26 | 0.000000 |
| Upregulated |  |  |  |
| CCND1 | NM_053056 | 2.39 | 0.000000 |
| CCNE1 | NM_001238 | 2.68 | 0.000000 |
| CTNNB1 | NM_001904 | 2.04 | 0.000000 |
| FOSL1 | NM_005438 | 2.05 | 0.000000 |
| HEYL | NM_014571 | 2.01 | 0.000000 |
| LMO2 | NM_005574 | 2.37 | 0.000000 |
| MMP7 | NM_002423 | 15.46 | 0.000000 |
| NCSTN | NM_015331 | 2.06 | 0.000000 |
| NFKB2 | NM_002502 | 6.55 | 0.000000 |
| PPARG | NM_015869 | 4.06 | 0.000000 |
| SH2D1A | NM_002351 | 4.44 | 0.000000 |
| WISP1 | NM_003882 | 3.65 | 0.000000 |

**Table S3. Primer sequences used to amplify specific target genes**

| Gene |  | Sequences(5’-3’) |
| --- | --- | --- |
| β-actin | Sense | GTTGCGTTACACCCTTTCTTG |
|  | Antisense | GACTGCTGTCACCTTCACCGT |
| MSI2 | Sense | ACCTCACCAGATAGCCTTAGAG |
|  | Antisense | AGCGTTTCGTAGTGGGATCTC |
| Nanog | Sense  Antisense | CCCCAGCCTTTACTCTTCCTA  CCAGGTTGAATTGTTCCAGGTC |
| Oct | Sense  Antisense | CTTGAATCCCGAATGGAAAGGG  GTGTATATCCCAGGGTGATCCTC |
| Sox2 | Sense  Antisense | GCCCTGCAGTACAACTCCAT  GACTTGACCACCGAACCCAT |
| LFNG | Sense  Antisense | GTCAGCGAGAACAAGGTGC  GATCCGCTCAGCCGTATTCAT |
| HR | Sense | AGGAGGCCATGCTTACCCAT |
|  | Antisense | AGACACTAGGTAGGGTGGCAA |
| STAT6 | Sense | GTTCCGCCACTTGCCAATG |
|  | Antisense | TGGATCTCCCCTACTCGGTG |
| FOS | Sense | CCGGGGATAGCCTCTCTTACT |
|  | Antisense | CCAGGTCCGTGCAGAAGTC |
| MMP7 | Sense | GAGTGAGCTACAGTGGGAACA |
|  | Antisense | CTATGACGCGGGAGTTTAACAT |
| NFKB2 | Sense | ATGGAGAGTTGCTACAACCCA |
|  | Antisense | CTGTTCCACGATCACCAGGTA |
| PPARG | Sense | GGGATCAGCTCCGTGGATCT |
|  | Antisense | TGCACTTTGGTACTCTTGAAGTT |
| SH2D1A | Sense | AGGCGTGTACTGCCTATGTG |
|  | Antisense | TGCAGAGGTATTACAATGCCTTG |
| WISP1 | Sense | GTGCTGTAAGATGTGCGCTCA |
|  | Antisense | CCCGCTGTAGTCACAGTAGAG |
